# Comparison of treatment models for single primary advanced gallbladder cancer

**DOI:** 10.3389/fimmu.2024.1500091

**Published:** 2024-11-13

**Authors:** Rongxuan Li, Xiao Chen, Bingchen Wang, Bolun Ai, Fangdi Min, Dayong Cao, Jianguo Zhou, Tao Yan

**Affiliations:** ^1^ Department of Hepatobiliary Surgery, National Cancer Center/National Clinical Research Center for Cancer/Cancer Hospital, Chinese Academy of Medical Sciences and Peking Union Medical College, Beijing, China; ^2^ Department of Breast Surgical Oncology, National Cancer Center/National Clinical Research Center for Cancer/Cancer Hospital, Chinese Academy of Medical Sciences and Peking Union Medical College, Beijing, China; ^3^ Department of Anesthesiology, National Cancer Center/National Clinical Research Center for Cancer/Cancer Hospital, Chinese Academy of Medical Sciences and Peking Union Medical College, Beijing, China

**Keywords:** advanced gallbladder cancer, surgery, chemotherapy, radiotherapy, immunotherapy, SEER, cancer-specific survival

## Abstract

**Purpose:**

Treatment for advanced gallbladder cancer (GBC) remains controversial, with various recommendations regarding the choice and combination of surgery and adjuvant therapy. The present article is targeting for the exploration of optimal treatment models for advanced GBC.

**Methods:**

AJCC (American Joint Committee on Cancer, 8th edition) stage III and stage IV GBC, were defined as advanced GBC. Patients with advanced GBC were identified using the Surveillance, Epidemiology, and End Results (SEER) database and departmental cohort. Because of the most representative, only gallbladder adenocarcinoma (GBAC) patients were selected. Based on their surgical status (No, Non-radical and Radical surgery), chemotherapy status (Chemotherapy, No chemotherapy), and radiotherapy status (Radiotherapy, No radiotherapy), treatment models were categorized. For the purposes of evaluating the treatment outcomes of various treatment models and determining the risk element for cancer-specific survival (CSS), Cox regression analysis was applied. Kaplan-Meier curves were used before and after adjusting for covariates, with log-rank tests used to analyze discrepancies between curves. Immunotherapy was analyzed using clinical data from departmental cohort. Finally, to compensate for the limitations of the database, a review examines the progress in treatment models for advanced GBC.

**Results:**

5,154 patients aged over 18 years with solitary primary advanced GBC were identified from the SEER database. In advanced GBC patients, the treatment model has emerged as a significant prognostic factor. “Radical surgery + Chemotherapy + Radiotherapy” models maximally improved the CSS of advanced GBC before and after adjusting for covariates, while “No surgery + No chemotherapy + No radiotherapy” model had the lowest CSS. The present conclusions were supported even after subgroup analysis by AJCC stage. The efficacy of immunotherapy was demonstrated in the departmental cohort analysis. Additionally, this article provides a comprehensive overview of recent advancements in various emerging treatment strategies.

**Conclusion:**

Even when optimal treatment model cannot be pursued, providing comprehensive combinations of treatments to advanced GBC patients whenever possible is always beneficial for their survival.

## 1 Introduction

As the most familiar cancer from biliary tract, GBC ranks 23rd among all tumors and 6th among digestive system tumors (1). The global morbidity and mortality of GBC have been increasing annually, Eastern Asia, South America, and Melanesia were the regions with highest ranking (2, 3), the latest global incidence and mortality data for GBC are visualized in **Supplementary Figure 1**. Moreover, the etiology of GBC varies across different countries (4). GBC is one of the few cancers that shows a gender difference (5), with the morbidity in females nearly three times that of males (6), and is the only digestive system tumor that is predominantly female (7). As early symptoms are rare and lymph nodes and distant metastases often occur early, three-quarters of GBC patients are diagnosed with advanced stages or metastases, which results in poor prognoses (5, 8). In fact, symptomatic gallstones lead to cholecystectomy in most cases of GBC (9, 10). Additionally, among the biliary tract cancers, GBC has the shortest median survival rate. Despite improvements in diagnosis and treatment over the years, its 5-year survival rate remains lower than 20% (3). Therefore, addressing the treatment of GBC, a highly lethal tumor, is a significant challenge worldwide, and there is still considerable controversy surrounding the treatment of advanced GBC patients. Given that adenocarcinoma is the most prevalent and representative pathological type of GBC (11), and that treatment research has primarily centered on gallbladder adenocarcinoma (GBAC) (12, 13), the present study specifically addresses GBAC to ensure homogeneity, as other histological types exhibit different biological behaviors and treatment responses (14). Currently, among all treatment options for gallbladder cancer (GBC), only surgical intervention has demonstrated clear effectiveness, while other options, including radiotherapy, chemotherapy, and immunotherapy, remain in the exploratory stage (15). In the present article the SEER database, departmental cohort and the PubMed database were utilized to investigate treatment models for patients with advanced GBC, while also summarizing alternative treatment options.

## 2 Patients and methods

### 2.1 Patients selection

**Figure 1:**
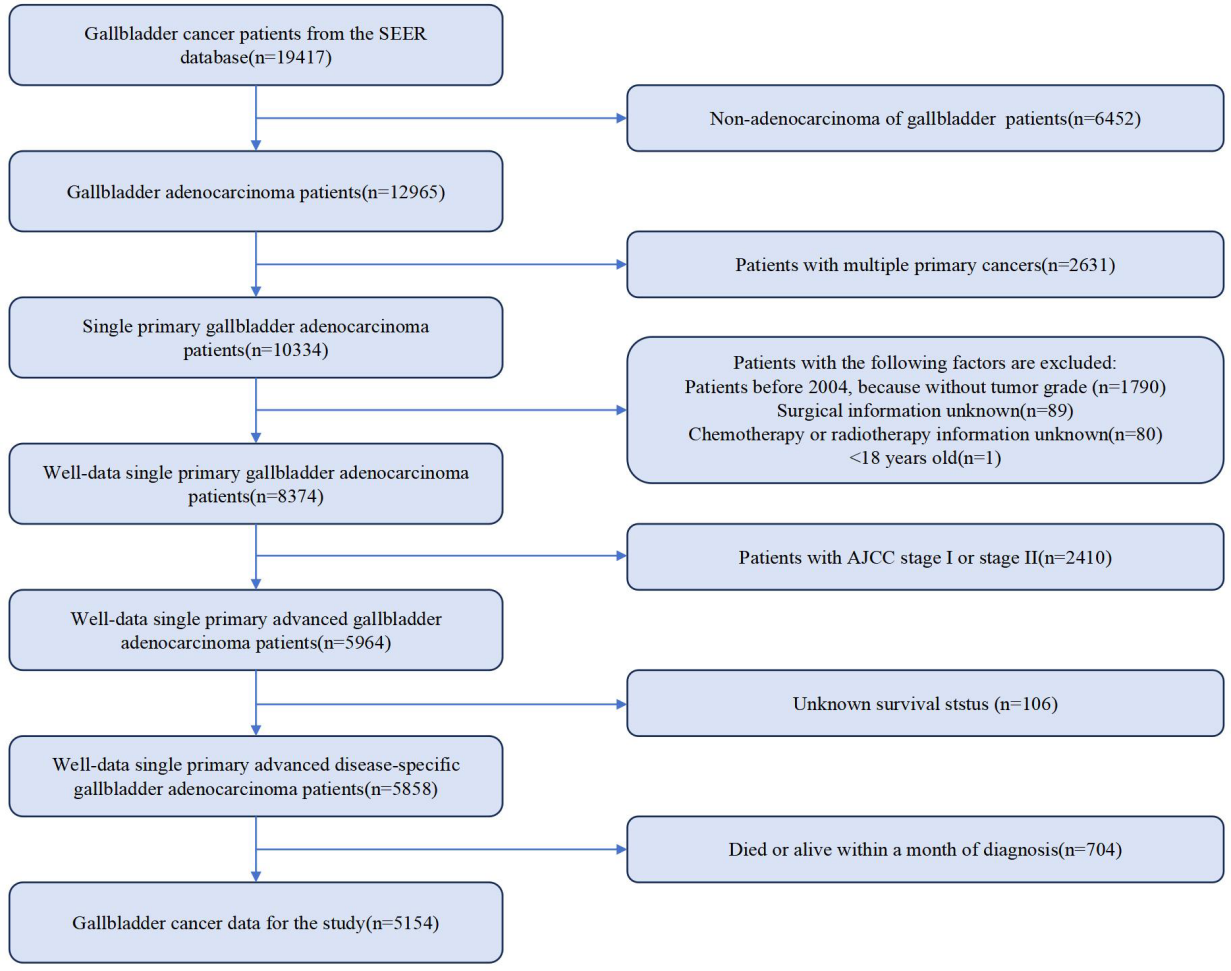
Flowchart of patient enrollment.

Information on demographic, cancer, treatment and follow-up is provided by the SEER database. To retrospectively collect GBC patient data, SEERStat (version 8.4.3) was used. 19,417 patients diagnosed with GBC from 2000 to 2019 were identified. Inclusion criteria were defined as Site recode ICD-O-3/WHO 2008 registered as gallbladder. Only those with AJCC stage III and stage IV GBC were selected. As per the AJCC, 8th edition, stage IIIA, IIIB, IVA and IVB were defined as “T3 + N0 + M0”, “T1-3 + N1 + M0”, “T4 + N0-1 + M0” and “Any T + N2 + M0” or “Any T + Any N + M1”, respectively. Exclusion criteria were as follows: not adenocarcinoma, not single primary cancer, patients diagnosed before 2004 (without tumor grade), unknown surgical information, without chemotherapy or radiotherapy information, less than 18 years old, AJCC stage I or stage II, unknown survival status, and death or alive within 1 month of diagnosis. Ultimately, 5,154 eligible patients with advanced GBC diagnosis remained. The detailed flowchart is illustrated in **Figure 1**. Given that immunotherapy is a novel treatment option, we selected only patients who underwent treatment in our department in 2022 for this study, with follow-up completed by July 30, 2023. CSS was recorded. The inclusion and exclusion criteria were identical to those used in SEER database cases. A total of 15 patients with complete data were included in the cohort, with demographic and clinical information presented in **Supplementary Table 1**.

### 2.2 Study covariates

The definitions and information on covariates such as age, gender, race, marital status, size, grade, T staging, N staging, M staging, surgical information, radiotherapy information, chemotherapy information and survival status were obtained in the SEER database. Deaths from GBC were taken as events, and survivors were censored based on CSS. For age at diagnosis, according to the WHO elderly classification standards, patients were classified into under 65 years old group and group for 65 years old and above. Gender included female and male. Races of patients were categorized as White, Black, Asian or Pacific Islander, American Indian or Alaska Native, and unknown. On the basis of marital status, patients were classified into groups with partners (Yes), without partners (No), and unknown marital status (Unknown). The without partners group includes single (never married), separated, divorced, and widowed, while for the with partners group, both same-sex and heterosexual couples were included. Tumor grades were classified as well, moderately, poorly and un- differentiated, corresponding to Grades I to IV, respectively, with the remaining patients labeled as Unknown grade. The optimal cutoff points determined by the X-tile software (version 3.6.1) were used to sort the tumors into groups of size <39, 40-60, ≥61, and unknown groups, with measurements in millimeters (mm). A screenshot of the software is presented as **Supplementary Figure 2**. T, N, and M staging were corrected based on the AJCC, 8th edition, and AJCC stages were generated. In surgical types, besides radical surgery, all other types of surgery were defined as non-radical surgery, with a separate group for no surgery performed. In the radiotherapy status, “None/Unknown” and “Refused” were considered as not received radiotherapy, while the rest were considered as received radiotherapy. In the chemotherapy status, “No/Unknown” was considered as not received chemotherapy, and “yes” was considered as received chemotherapy. Survival status included alive and dead. The data in the departmental cohort were categorized in the same manner.

### 2.3 Statistical analysis

The quantity (percentage (%)) was used to specify the categorical variables. Pearson’s Chi-squared test and Fisher’s exact test were employed to describe baseline characteristics. Cox proportional hazards models were used to evaluate the impact of covariates on the risk of CSS, and calculate the hazard ratios (HR) and 95% confidence intervals (CI) for advanced GBC. CSS was predicted using Kaplan-Meier curves before and after adjusting for covariates in different models, with log-rank tests used to analyze discrepancies between curves. Cramér’s V analysis was applied to assess the correlation between covariates. Propensity score matching (PSM) was used to match demographic baseline statistical characteristics. P-values < 0.05 is considered statistically significant. By using MSTATA software (https://www.mstata.com/), all analyses were carried out utilizing the R software (version 4.2.2) for statistical computing. Ultimately, through a comprehensive search of the literature database, we supplemented the treatment options not addressed in the SEER database and synthesized these findings to enhance the interpretation of our conclusions.

## 3 Results

### 3.1 Demographic and clinical characteristics

Altogether 5,154 eligible GBC patients were involved in the study cohort from 2000 to 2019. Among them, there were 849 cases with “No surgery + No chemotherapy + No radiotherapy” (NSNCNR) model, 1109 cases with “No surgery + Chemotherapy + No radiotherapy” (NSCNR) model, 45 cases with “No surgery + No chemotherapy + Radiotherapy” (NSNCR) model, 132 cases with “No surgery + Chemotherapy + Radiotherapy” (NSCR) model, 1185 cases with “Non-radical surgery + No chemotherapy + No radiotherapy” (NrSNCNR) model, 882 cases with “Non-radical surgery + Chemotherapy + No radiotherapy” (NrSCNR) model, 83 cases with “Non-radical surgery + No chemotherapy + Radiotherapy” (NrSNCR) model, 411 cases with “Non-radical surgery + Chemotherapy + Radiotherapy” (NrSCR) model, 190 cases with “Radical surgery + No chemotherapy + No radiotherapy” (RSNCNR) model, 131 cases with “Radical surgery + Chemotherapy + No radiotherapy” (RSCNR) model, 12 cases with “Radical surgery + No chemotherapy + Radiotherapy” (RSNCR) model, and 125 cases with “Radical surgery + Chemotherapy + Radiotherapy” (RSCR) model. For ease of reading, these treatment models are clearly designated by the letters A to L in the tables and figures. According to **Table 1**, the overall and different treatment models for GBC patients differ in demographics and clinical characteristics. Most patients were aged 65 or older (61.37%), and the distribution by gender was skewed toward females (70.70%). The racial composition was predominantly White (75.07%), followed by Black (12.44%) and Asian or Pacific Islander (11.08%). Over half of the participants were married (51.96%). The distribution by tumor size varied, with 27.90% having a tumor size of 39 or less, 14.82% between 40-60, and 11.91% greater than or equal to 61, with measurements in millimeters (mm). Grade I, II, III, IV, and grade Unknown accounted for 4.89%, 24.64%, 27.69%, 0.91%, and 41.87%, respectively. For AJCC stage perspective, stage IVB held the highest percentage of participants (56.67%). Various treatment models were administered, with the most common being “NrSNCNR” (23.00%), “NSCNR” (21.52%), “NrSCNR” (17.11%), and “NSNCNR” (16.47%). 81.99% of participants were dead when assessment. The line chart illustrating the selection of various treatment models over time is presented in **Supplementary Figure 3**.

**Table 1:** Demographic and clinical characteristics of advanced GBC patients receiving twelve treatment models.

Characteristic	Treatment Model												
	All, N = 5,154	A, N = 849^1^	B, N = 1,109^1^	C, N = 45^1^	D, N = 132^1^	E, N = 1,185^1^	F, N = 882^1^	G, N = 83^1^	H, N = 411^1^	I, N = 190^1^	J, N = 131^1^	K, N = 12^1^	L, N = 125^1^
Age													
<65	1,991 (38.63%)	235 (27.68%)	532 (47.97%)	15 (33.33%)	58 (43.94%)	283 (23.88%)	438 (49.66%)	26 (31.33%)	201 (48.91%)	61 (32.11%)	65 (49.62%)	5 (41.67%)	72 (57.60%)
≥65	3,163 (61.37%)	614 (72.32%)	577 (52.03%)	30 (66.67%)	74 (56.06%)	902 (76.12%)	444 (50.34%)	57 (68.67%)	210 (51.09%)	129 (67.89%)	66 (50.38%)	7 (58.33%)	53 (42.40%)
Gender													
Female	3,644 (70.70%)	596 (70.20%)	735 (66.28%)	34 (75.56%)	88 (66.67%)	882 (74.43%)	642 (72.79%)	63 (75.90%)	295 (71.78%)	127 (66.84%)	94 (71.76%)	9 (75.00%)	79 (63.20%)
Male	1,510 (29.30%)	253 (29.80%)	374 (33.72%)	11 (24.44%)	44 (33.33%)	303 (25.57%)	240 (27.21%)	20 (24.10%)	116 (28.22%)	63 (33.16%)	37 (28.24%)	3 (25.00%)	46 (36.80%)
Race													
White	3,869 (75.07%)	611 (71.97%)	794 (71.60%)	26 (57.78%)	95 (71.97%)	945 (79.75%)	702 (79.59%)	58 (69.88%)	304 (73.97%)	138 (72.63%)	91 (69.47%)	9 (75.00%)	96 (76.80%)
Black	641 (12.44%)	129 (15.19%)	170 (15.33%)	8 (17.78%)	18 (13.64%)	102 (8.61%)	99 (11.22%)	11 (13.25%)	48 (11.68%)	25 (13.16%)	16 (12.21%)	2 (16.67%)	13 (10.40%)
Asian or Pacific Islander	571 (11.08%)	94 (11.07%)	130 (11.72%)	9 (20.00%)	17 (12.88%)	123 (10.38%)	73 (8.28%)	14 (16.87%)	54 (13.14%)	20 (10.53%)	23 (17.56%)	1 (8.33%)	13 (10.40%)
American Indian/ Alaska Native	59 (1.14%)	12 (1.41%)	11 (0.99%)	2 (4.44%)	2 (1.52%)	13 (1.10%)	6 (0.68%)	0 (0.00%)	4 (0.97%)	6 (3.16%)	1 (0.76%)	0 (0.00%)	2 (1.60%)
Unknown	14 (0.27%)	3 (0.35%)	4 (0.36%)	0 (0.00%)	0 (0.00%)	2 (0.17%)	2 (0.23%)	0 (0.00%)	1 (0.24%)	1 (0.53%)	0 (0.00%)	0 (0.00%)	1 (0.80%)
Marital													
Yes	2,678 (51.96%)	349 (41.11%)	651 (58.70%)	23 (51.11%)	74 (56.06%)	504 (42.53%)	508 (57.60%)	51 (61.45%)	256 (62.29%)	91 (47.89%)	78 (59.54%)	6 (50.00%)	87 (69.60%)
No	2,305 (44.72%)	461 (54.30%)	431 (38.86%)	20 (44.44%)	57 (43.18%)	640 (54.01%)	337 (38.21%)	30 (36.14%)	141 (34.31%)	95 (50.00%)	52 (39.69%)	5 (41.67%)	36 (28.80%)
Unknown	171 (3.32%)	39 (4.59%)	27 (2.43%)	2 (4.44%)	1 (0.76%)	41 (3.46%)	37 (4.20%)	2 (2.41%)	14 (3.41%)	4 (2.11%)	1 (0.76%)	1 (8.33%)	2 (1.60%)
Size													
≤39	1,438 (27.90%)	122 (14.37%)	163 (14.70%)	5 (11.11%)	18 (13.64%)	435 (36.71%)	347 (39.34%)	28 (33.73%)	171 (41.61%)	57 (30.00%)	33 (25.19%)	7 (58.33%)	52 (41.60%)
40-60	764 (14.82%)	90 (10.60%)	131 (11.81%)	9 (20.00%)	27 (20.45%)	174 (14.68%)	133 (15.08%)	14 (16.87%)	76 (18.49%)	34 (17.89%)	41 (31.30%)	1 (8.33%)	34 (27.20%)
≥61	614 (11.91%)	128 (15.08%)	155 (13.98%)	5 (11.11%)	20 (15.15%)	115 (9.70%)	79 (8.96%)	2 (2.41%)	31 (7.54%)	42 (22.11%)	21 (16.03%)	2 (16.67%)	14 (11.20%)
Unknown	2,338 (45.36%)	509 (59.95%)	660 (59.51%)	26 (57.78%)	67 (50.76%)	461 (38.90%)	323 (36.62%)	39 (46.99%)	133 (32.36%)	57 (30.00%)	36 (27.48%)	2 (16.67%)	25 (20.00%)
Grade													
Grade I	252 (4.89%)	14 (1.65%)	12 (1.08%)	2 (4.44%)	2 (1.52%)	103 (8.69%)	47 (5.33%)	8 (9.64%)	38 (9.25%)	12 (6.32%)	6 (4.58%)	2 (16.67%)	6 (4.80%)
Grade II	1,270 (24.64%)	69 (8.13%)	91 (8.21%)	1 (2.22%)	12 (9.09%)	431 (36.37%)	279 (31.63%)	25 (30.12%)	181 (44.04%)	70 (36.84%)	48 (36.64%)	5 (41.67%)	58 (46.40%)
Grade III	1,427 (27.69%)	106 (12.49%)	132 (11.90%)	9 (20.00%)	23 (17.42%)	462 (38.99%)	348 (39.46%)	43 (51.81%)	137 (33.33%)	77 (40.53%)	47 (35.88%)	5 (41.67%)	38 (30.40%)
Grade IV	47 (0.91%)	2 (0.24%)	6 (0.54%)	0 (0.00%)	0 (0.00%)	15 (1.27%)	9 (1.02%)	2 (2.41%)	6 (1.46%)	3 (1.58%)	3 (2.29%)	0 (0.00%)	1 (0.80%)
Unknown	2,158 (41.87%)	658 (77.50%)	868 (78.27%)	33 (73.33%)	95 (71.97%)	174 (14.68%)	199 (22.56%)	5 (6.02%)	49 (11.92%)	28 (14.74%)	27 (20.61%)	0 (0.00%)	22 (17.60%)
Stage													
IIIA	1,260 (24.45%)	160 (18.85%)	135 (12.17%)	10 (22.22%)	35 (26.52%)	472 (39.83%)	184 (20.86%)	32 (38.55%)	108 (26.28%)	57 (30.00%)	26 (19.85%)	5 (41.67%)	36 (28.80%)
IIIB	809 (15.70%)	6 (0.71%)	8 (0.72%)	1 (2.22%)	1 (0.76%)	241 (20.34%)	193 (21.88%)	26 (31.33%)	191 (46.47%)	51 (26.84%)	31 (23.66%)	6 (50.00%)	54 (43.20%)
IVA	164 (3.18%)	34 (4.00%)	38 (3.43%)	1 (2.22%)	15 (11.36%)	23 (1.94%)	15 (1.70%)	2 (2.41%)	8 (1.95%)	10 (5.26%)	10 (7.63%)	0 (0.00%)	8 (6.40%)
IVB	2,921 (56.67%)	649 (76.44%)	928 (83.68%)	33 (73.33%)	81 (61.36%)	449 (37.89%)	490 (55.56%)	23 (27.71%)	104 (25.30%)	72 (37.89%)	64 (48.85%)	1 (8.33%)	27 (21.60%)
Status													
Dead	4,226 (81.99%)	765 (90.11%)	968 (87.29%)	41 (91.11%)	117 (88.64%)	955 (80.59%)	677 (76.76%)	69 (83.13%)	299 (72.75%)	141 (74.21%)	93 (70.99%)	11 (91.67%)	90 (72.00%)
Alive	928 (18.01%)	84 (9.89%)	141 (12.71%)	4 (8.89%)	15 (11.36%)	230 (19.41%)	205 (23.24%)	14 (16.87%)	112 (27.25%)	49 (25.79%)	38 (29.01%)	1 (8.33%)	35 (28.00%)

^1^n (%).

A:No surgery + No chemotherapy + No radiotherapy; B:No surgery + Chemotherapy + No radiotherapy; C:No surgery + No chemotherapy + Radiotherapy; D:No surgery + Chemotherapy + Radiotherapy; E:Non-radical surgery + No chemotherapy + No radiotherapy; F:Non-radical surgery + Chemotherapy + No radiotherapy; G:Non-radical surgery + No chemotherapy + Radiotherapy; H:Non-radical surgery + Chemotherapy + Radiotherapy; I:Radical surgery + No chemotherapy + No radiotherapy; J:Radical surgery + Chemotherapy + No radiotherapy; K:Radical surgery + No chemotherapy + Radiotherapy; L:Radical surgery + Chemotherapy + Radiotherapy.

### 3.2 Identification of risk factors

Univariate Cox regression analysis indicated that age, gender, marital status, tumor size, tumor grade, AJCC stage, and treatment model significantly influenced CSS in advanced GBC patients, while race did not show a significant impact. After removing covariates with nonsignificant (p > 0.05) effects on survival time from the univariate Cox regression analysis, **Table 2** presents the results for the remaining covariates by multivariate Cox regression analysis. In our analysis, the covariates did not exhibit significant collinearity, as detailed in **Supplementary Figure 4**.

**Table 2:** Univariate and multivariate Cox proportional hazards models of CSS for advanced GBC patients in twelve treatment models.

Characteristic	Univariable					Multivariable				
	N	Event N	HR^1^	95% CI^1^	p-value	N	Event N	HR^1^	95% CI^1^	p-value
Age										
<65	1,991	1,608	reference	reference		1,991	1,608	reference	reference	
≥65	3,163	2,618	1.22	1.14, 1.29	<0.001	3,163	2,618	1.14	1.07, 1.21	<0.001
Gender										
Female	3,644	2,959	reference	reference		3,644	2,959	reference	reference	
Male	1,510	1,267	1.1	1.03, 1.17	0.006	1,510	1,267	1.11	1.04, 1.19	0.002
Race										
White	3,869	3,162	reference	reference						
Black	641	536	1.08	0.99, 1.19	0.084					
Asian or Pacific Islander	571	473	1	0.91, 1.10	0.985					
American Indian/Alaska Native	59	49	1.32	1.00, 1.76	0.051					
Unknown	14	6	0.53	0.24, 1.19	0.123					
Marital										
Yes	2,678	2,189	reference	reference		2,678	2,189	reference	reference	
No	2,305	1,898	1.17	1.10, 1.24	<0.001	2,305	1,898	1.1	1.03, 1.17	0.005
Unknown	171	139	1.11	0.93, 1.31	0.242	171	139	1.19	1.00, 1.42	0.046
Size										
≤39	1,438	1,094	reference	reference		1,438	1,094	reference	reference	
40-60	764	603	1.21	1.10, 1.34	<0.001	764	603	1.12	1.01, 1.24	0.031
≥61	614	514	1.67	1.51, 1.86	<0.001	614	514	1.39	1.25, 1.54	<0.001
Unknown	2,338	2,015	1.65	1.53, 1.78	<0.001	2,338	2,015	1.23	1.14, 1.33	<0.001
Grade										
Grade I	252	196	reference	reference		252	196	reference	reference	
Grade II	1,270	1,042	1.17	1.00, 1.36	0.045	1,270	1,042	1.26	1.08, 1.47	0.003
Grade III	1,427	1,240	1.68	1.45, 1.96	<0.001	1,427	1,240	1.74	1.50, 2.03	<0.001
Grade IV	47	40	1.97	1.40, 2.77	<0.001	47	40	2.3	1.63, 3.24	<0.001
Unknown	2,158	1,708	2.23	1.92, 2.59	<0.001	2,158	1,708	1.43	1.22, 1.67	<0.001
Stage										
IIIA	1,260	993	reference	reference		1,260	993	reference	reference	
IIIB	809	520	0.57	0.51, 0.63	<0.001	809	520	0.74	0.66, 0.82	<0.001
IVA	164	141	1.34	1.13, 1.60	0.001	164	141	1.31	1.10, 1.57	0.003
IVB	2,921	2,572	1.78	1.65, 1.92	<0.001	2,921	2,572	1.75	1.62, 1.90	<0.001
Treatment										
A	849	765	reference	reference		849	765	reference	reference	
B	1,109	968	0.46	0.41, 0.50	<0.001	1,109	968	0.43	0.39, 0.48	<0.001
C	45	41	0.75	0.55, 1.03	0.077	45	41	0.83	0.61, 1.14	0.256
D	132	117	0.4	0.33, 0.48	<0.001	132	117	0.42	0.35, 0.51	<0.001
E	1,185	955	0.34	0.31, 0.38	<0.001	1,185	955	0.49	0.44, 0.55	<0.001
F	882	677	0.24	0.22, 0.27	<0.001	882	677	0.3	0.27, 0.34	<0.001
G	83	69	0.26	0.20, 0.33	<0.001	83	69	0.38	0.30, 0.50	<0.001
H	411	299	0.16	0.14, 0.18	<0.001	411	299	0.25	0.21, 0.29	<0.001
I	190	141	0.28	0.23, 0.33	<0.001	190	141	0.38	0.31, 0.46	<0.001
J	131	93	0.21	0.17, 0.26	<0.001	131	93	0.25	0.20, 0.32	<0.001
K	12	11	0.21	0.12, 0.39	<0.001	12	11	0.37	0.20, 0.68	0.001
L	125	90	0.15	0.12, 0.19	<0.001	125	90	0.24	0.19, 0.31	<0.001

^1^HR, Hazard Ratio; CI, Confidence Interval.

**Figure 2:**
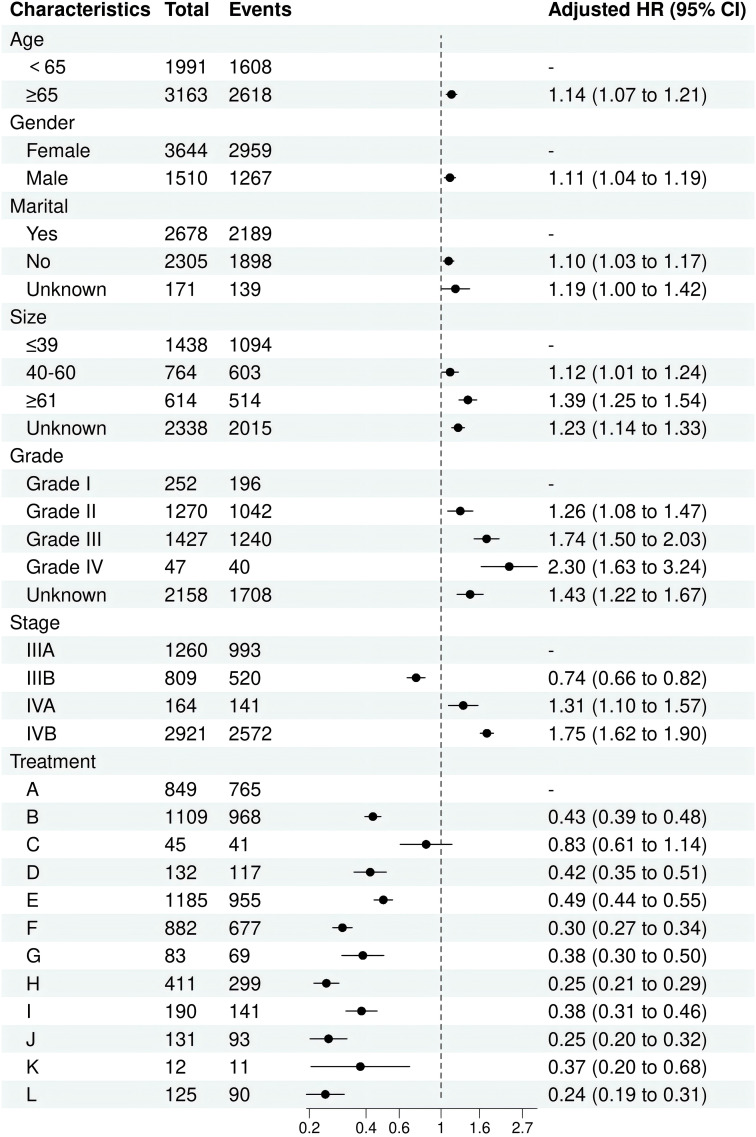
The forest plot for multivariate Cox proportional hazards models for CSS in twelve treatment models.

In line with the Cox regression analysis, the following treatment models had significantly lower hazard ratios (HR) for CSS than “NSNCNR”: “NSCNR” (HR = 0.43, 95% CI 0.39-0.48, p < 0.001), “NSNCR” (HR = 0.83, 95% CI 0.61-1.14, p = 0.256), “NSCR” (HR = 0.42, 95% CI 0.35-0.51, p < 0.001), “NrSNCNR” (HR = 0.49, 95% CI 0.44-0.55, p < 0.001), “NrSCNR” (HR = 0.30, 95% CI 0.27-0.34, p < 0.001), “NrSNCR” (HR = 0.38, 95% CI 0.30-0.50, p < 0.001), “NrSCR” (HR = 0.25, 95% CI 0.21-0.29, p < 0.001), “RSNCNR” (HR = 0.38, 95% CI 0.31-0.46, p < 0.001), “RSCNR” (HR = 0.25, 95% CI 0.20-0.32, p < 0.001), “RSNCR” (HR = 0.37, 95% CI 0.20-0.68, p = 0.001), and “RSCR” (HR = 0.24, 95% CI 0.19-0.31, p < 0.001). The forest plot was shown in **Figure 2**. As a supplement, the results of univariate and multivariate analyses for the remaining treatment models, after excluding those with insufficient sample sizes (<1%), have been added to **Supplementary Table 2**. Additionally, to eliminate baseline differences between groups, PSM was applied based on whether patients received radical or non-radical surgery. The resulting baseline table and the Cox regression analysis have been designated as **Supplementary Tables 3**, **4**, respectively. Due to missing data and a large number of “Unknown” entries, **Supplementary Table 5** was created based on patients with complete data. The findings indicated that the results presented in the **Supplementary Material** showed trends that are nearly identical to those from the original analysis. Therefore, we opted to present the results from the original cohort, which includes a larger number of patients, in the main text.

### 3.3 Survival curve of each treatment group

**Figure 3:**
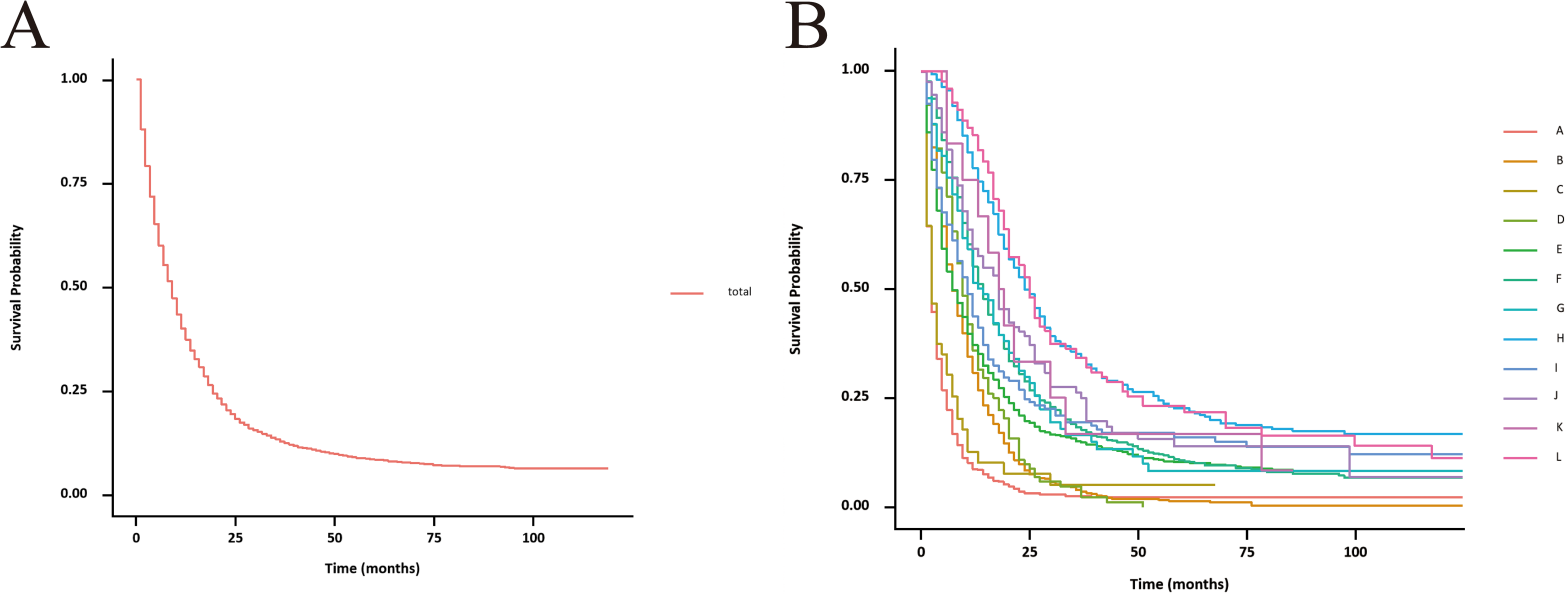
Unadjusted CSS curves for advanced GBC patients. **(A)**, total patients; **(B)**, twelve treatment models.

**Figure 4:**
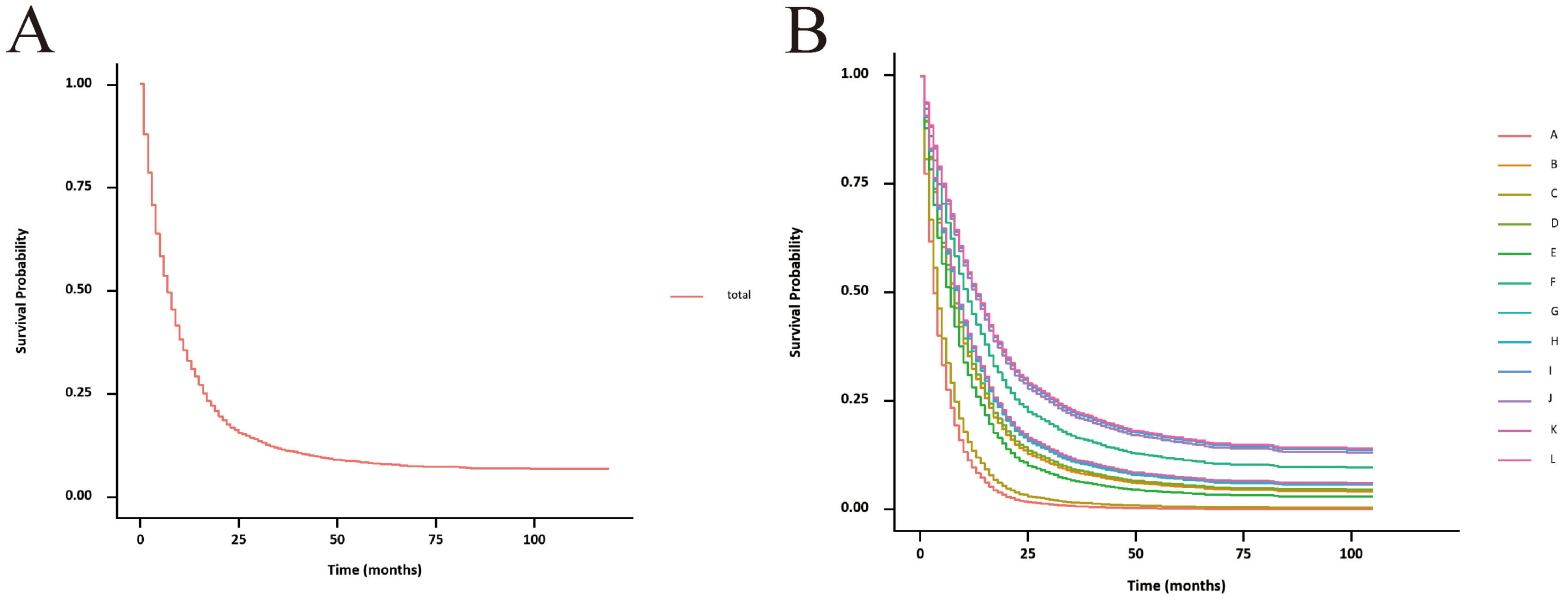
Adjusted CSS curves for advanced GBC patients. **(A)**, total patients; **(B)**, twelve treatment models.

The unadjusted survival curves for advanced GBC patients as a whole and for each treatment model were shown in **Figure 3**. After adjusting the covariates that were significant in multivariate Cox regression analysis, the CSS curves with advanced GBC and each treatment model were shown in **Figure 4**. The adjusted survival curves were calculated and plotted using the “conditional” method, which was based on the Cox proportional hazards models (16). This method involves creating multiple copies of the data to balance covariate differences across groups, resulting in a more accurate assessment of the effect of group membership on survival outcomes.

### 3.4 Subgroup analysis

**Figure 5:**
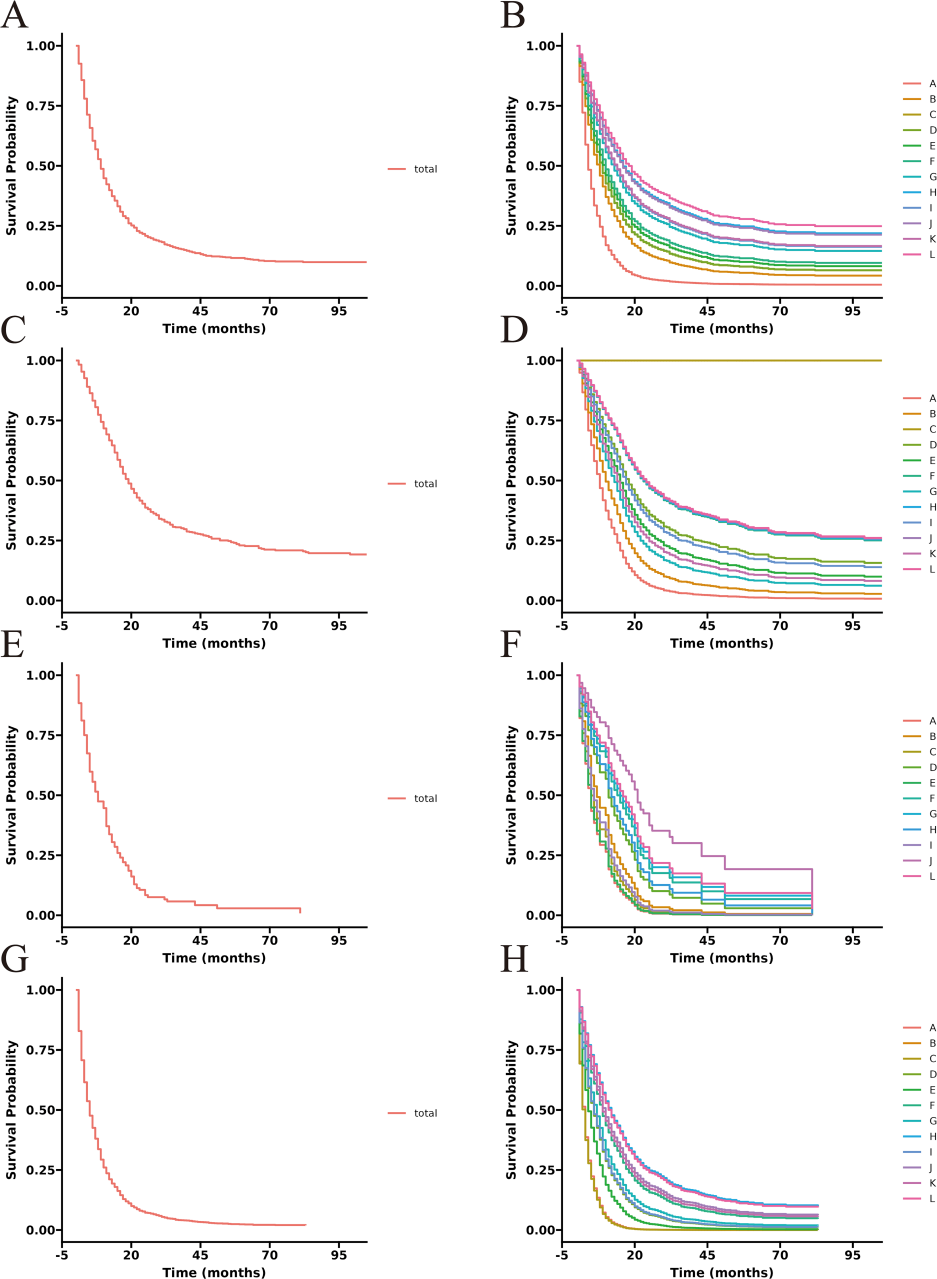
Adjusted CSS curves for advanced GBC patients at different pathological stages. Stage IIIA: **(A)**, total patients; **(B)**, twelve treatment models. Stage IIIB: **(C)**, total patients; **(D)**, twelve treatment models. Stage IVA: **(E)**, total patients; **(F)**, twelve treatment models. Stage IVB: **(G)**, total patients; **(H)**, twelve treatment models.

**Figure 6:**
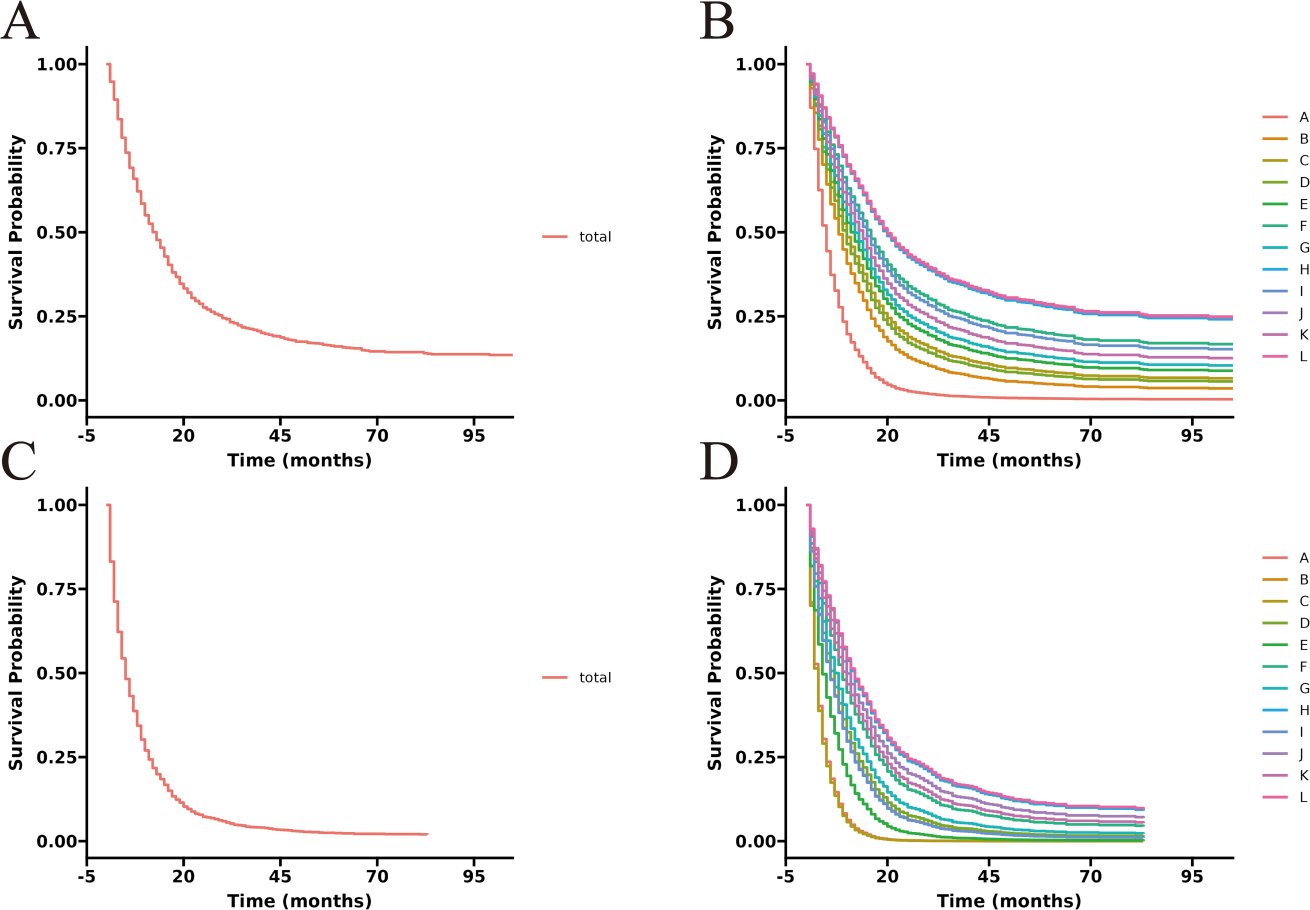
Adjusted CSS curves for advanced GBC patients at different pathological stages. Stage III: **(A)**, total patients; **(B)**, twelve treatment models. Stage IV: **(C)**, total patients; **(D)**, twelve treatment models.

To better demonstrate the impact of different treatment models on CSS for advanced GBC patients, we grouped all patients based on AJCC stage in addition to the multivariate Cox regression analysis. It was noticed that for advanced GBC patients, treatment model remained an independent prognostic factor in **Table 3** and **Figure 5**. “RSCR” was regarded as the most effective treatment models for CSS in AJCC stage IIIA, “RSCR” and “RSCNR” were identified as the most effective treatment models for CSS in AJCC stage IIIB, while “RSCNR” was found to be the most effective for AJCC stage IVA, “NrSCR” and “RSCR” were noticed as the most effective for AJCC stage IVB. Based on these speculations, patients were combined into AJCC stage III and stage IV to mitigate differences in patient numbers between different stages, and the results obtained were generally consistent with the previous findings in **Table 4** and **Figure 6**. It was noteworthy that regardless of being in AJCC stage III or stage IV, “RSCR” model exhibited the best HR values, followed by “NrSCR” and “RSCNR”.

**Table 3:** Multivariate Cox proportional hazards models of CSS for advanced GBC in twelve treatment models at different pathological stages, reported separately for stage IIIA, IIIB, IVA and IVB.

Characteristic	IIIA				IIIB				IVA				IVB			
	N	HR^1^	95% CI^1^	p-value	N	HR^1^	95% CI^1^	p-value	N	HR^1^	95% CI^1^	p-value	N	HR^1^	95% CI^1^	p-value
Age																
<65	398	reference	reference		294	reference	reference		65	reference	reference		1,234	reference	reference	
≥65	862	1.33	1.15, 1.54	<0.001	515	1.19	0.98, 1.44	0.08	99	1.38	0.89, 2.13	0.152	1,687	1.09	1.01, 1.18	0.035
Gender																
Female	878	reference	reference		591	reference	reference		114	reference	reference		2,061	reference	reference	
Male	382	1.11	0.96, 1.28	0.16	218	1.42	1.17, 1.74	<0.001	50	0.72	0.47, 1.12	0.145	860	1.1	1.00, 1.19	0.038
Marital																
Yes	607	reference	reference		445	reference	reference		85	reference	reference		1,541	reference	reference	
No	602	1.31	1.14, 1.50	<0.001	334	1.08	0.88, 1.31	0.458	73	1.08	0.74, 1.59	0.678	1,296	1.04	0.96, 1.13	0.298
Unknown	51	1.26	0.90, 1.76	0.183	30	1.12	0.71, 1.77	0.618	6	0.87	0.33, 2.28	0.782	84	1.26	0.99, 1.59	0.056
Size																
≤39	385	reference	reference		373	reference	reference		31	reference	reference		649	reference	reference	
40-60	204	1.19	0.97, 1.46	0.087	138	1.18	0.91, 1.53	0.206	35	1.11	0.61, 2.01	0.73	387	1.09	0.95, 1.26	0.2
≥61	184	1.6	1.30, 1.98	<0.001	71	2.14	1.58, 2.88	<0.001	25	1.05	0.55, 2.03	0.873	334	1.18	1.02, 1.36	0.025
Unknown	487	1.22	1.04, 1.43	0.015	227	1.26	1.02, 1.56	0.029	73	1.17	0.71, 1.94	0.538	1,551	1.18	1.07, 1.31	0.001
Grade																
Grade I	81	reference	reference		68	reference	reference		8	reference	reference		95	reference	reference	
Grade II	393	1.35	1.02, 1.78	0.039	301	1.21	0.87, 1.69	0.253	45	1.1	0.46, 2.66	0.83	531	1.21	0.96, 1.53	0.105
Grade III	401	1.87	1.41, 2.48	<0.001	272	1.62	1.16, 2.26	0.004	45	2.43	1.03, 5.71	0.042	709	1.62	1.29, 2.04	<0.001
Grade IV	11	2.97	1.49, 5.89	0.002	11	1.83	0.81, 4.13	0.144	1	0.51	0.06, 4.51	0.543	24	2.27	1.42, 3.64	<0.001
Unknown	374	1.54	1.14, 2.09	0.005	157	1.38	0.92, 2.08	0.124	65	1.32	0.52, 3.39	0.562	1,562	1.34	1.06, 1.68	0.013
Treatment																
A	160	reference	reference		6	reference	reference		34	reference	reference		649	reference	reference	
B	135	0.54	0.42, 0.70	<0.001	8	0.7	0.20, 2.44	0.581	38	0.63	0.35, 1.12	0.113	928	0.42	0.37, 0.46	<0.001
C	10	0.46	0.22, 0.95	0.037	1	0	0.00, Inf	0.991	1	0.82	0.11, 6.31	0.85	33	1.03	0.72, 1.48	0.853
D	35	0.46	0.31, 0.68	<0.001	1	0.34	0.04, 3.10	0.34	15	0.39	0.19, 0.81	0.011	81	0.42	0.33, 0.54	<0.001
E	472	0.42	0.33, 0.52	<0.001	241	0.43	0.16, 1.20	0.109	23	0.96	0.50, 1.84	0.896	449	0.57	0.49, 0.65	<0.001
F	184	0.39	0.30, 0.50	<0.001	193	0.25	0.09, 0.70	0.008	15	0.29	0.13, 0.65	0.003	490	0.28	0.24, 0.32	<0.001
G	32	0.31	0.20, 0.49	<0.001	26	0.53	0.18, 1.59	0.259	2	0.26	0.03, 2.06	0.203	23	0.37	0.24, 0.57	<0.001
H	108	0.24	0.18, 0.33	<0.001	191	0.25	0.09, 0.69	0.008	8	0.35	0.13, 0.91	0.032	104	0.21	0.16, 0.26	<0.001
I	57	0.29	0.20, 0.43	<0.001	51	0.37	0.13, 1.06	0.065	10	0.75	0.31, 1.85	0.537	72	0.41	0.31, 0.54	<0.001
J	26	0.25	0.14, 0.42	<0.001	31	0.24	0.08, 0.76	0.015	10	0.16	0.06, 0.42	<0.001	64	0.25	0.19, 0.34	<0.001
K	5	0.29	0.11, 0.80	0.016	6	0.47	0.13, 1.74	0.261					1	0.26	0.04, 1.89	0.184
L	36	0.22	0.14, 0.35	<0.001	54	0.24	0.08, 0.71	0.01	8	0.25	0.10, 0.61	0.003	27	0.21	0.14, 0.33	<0.001

^1^HR, Hazard Ratio; CI, Confidence Interval.

**Table 4:** Multivariate Cox proportional hazards models of CSS for advanced GBC in twelve treatment models at different pathological stages, reported separately for stage III and IV.

Characteristic	III				IV			
	N	HR^1^	95% CI^1^	p-value	N	HR^1^	95% CI^1^	p-value
Age								
<65	692	reference	reference		1,299	reference	reference	
≥65	1,377	1.28	1.14, 1.43	<0.001	1,786	1.09	1.01, 1.18	0.034
Gender								
Female	1,469	reference	reference		2,175	reference	reference	
Male	600	1.21	1.08, 1.36	0.001	910	1.07	0.98, 1.16	0.109
Marital								
Yes	1,052	reference	reference		1,626	reference	reference	
No	936	1.22	1.10, 1.37	<0.001	1,369	1.05	0.97, 1.13	0.254
Unknown	81	1.2	0.92, 1.57	0.177	90	1.21	0.97, 1.52	0.093
Size								
≤39	758	reference	reference		680	reference	reference	
40-60	342	1.2	1.03, 1.41	0.019	422	1.07	0.94, 1.22	0.322
≥61	255	1.8	1.52, 2.14	<0.001	359	1.17	1.01, 1.34	0.032
Unknown	714	1.28	1.13, 1.45	<0.001	1,624	1.19	1.07, 1.31	<0.001
Grade								
Grade I	149	reference	reference		103	reference	reference	
Grade II	694	1.29	1.04, 1.60	0.02	576	1.21	0.97, 1.51	0.094
Grade III	673	1.76	1.42, 2.18	<0.001	754	1.68	1.35, 2.09	<0.001
Grade IV	22	2.21	1.32, 3.71	0.003	25	2.11	1.33, 3.34	0.001
Unknown	531	1.42	1.12, 1.81	0.004	1,627	1.36	1.09, 1.70	0.006
Treatment								
A	166	reference	reference		683	reference	reference	
B	143	0.54	0.42, 0.69	<0.001	966	0.43	0.38, 0.48	<0.001
C	11	0.43	0.21, 0.88	0.021	34	1.04	0.73, 1.48	0.831
D	36	0.45	0.31, 0.67	<0.001	96	0.4	0.32, 0.50	<0.001
E	713	0.38	0.31, 0.46	<0.001	472	0.58	0.51, 0.67	<0.001
F	377	0.27	0.22, 0.34	<0.001	505	0.29	0.25, 0.33	<0.001
G	58	0.35	0.25, 0.49	<0.001	25	0.35	0.23, 0.55	<0.001
H	299	0.21	0.17, 0.27	<0.001	112	0.22	0.17, 0.28	<0.001
I	108	0.28	0.21, 0.38	<0.001	82	0.43	0.33, 0.56	<0.001
J	57	0.21	0.14, 0.31	<0.001	74	0.24	0.19, 0.32	<0.001
K	11	0.32	0.16, 0.61	<0.001	1	0.27	0.04, 1.92	0.19
L	90	0.21	0.15, 0.28	<0.001	35	0.21	0.15, 0.31	<0.001

^1^HR, Hazard Ratio; CI, Confidence Interval.

### 3.5 Departmental cohort analysis

**Figure 7:**
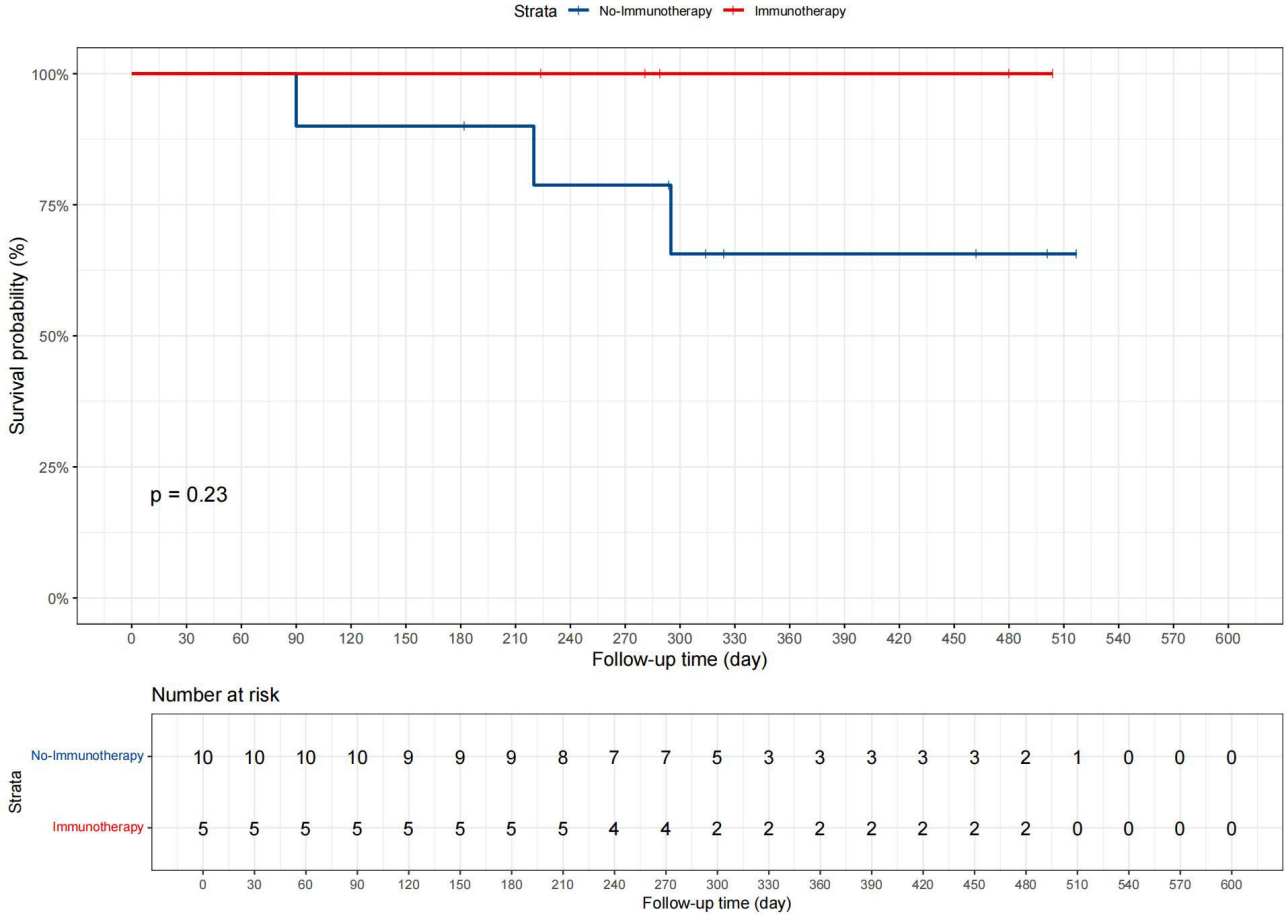
The CSS curves for advanced GBC patients in the departmental cohort.

Patients were divided into two groups based on whether they received immunotherapy: 5 in the “Immunotherapy” group and 10 in the “No immunotherapy” group. Data such as age, gender, and tumor size were included in the analysis, and the demographic and clinical characteristics are displayed in **Supplementary Table 6**. Statistical analysis revealed no significant differences between the groups for any measured variables, with all p-values greater than 0.05. Additionally, in the present study, we conducted Kaplan-Meier survival analysis, as shown in **Figure 7**, to compare CSS between patients who received immunotherapy and those who did not. The survival curves suggested a trend toward improved prognosis in the immunotherapy group, but the p-value from the log-rank test was 0.23.

## 4 Discussion

Currently, treatment options for advanced GBC patients remain uncertain. Surgery continues to be the preferred treatment option for advanced GBC. Radical surgical is the only potential curative method for advanced GBC patients. Unfortunately, due to lymph node and distant metastases in advanced GBC, which limit the opportunity for radical surgery, non-radical surgery, as an alternative, including procedures aimed at relieving jaundice or pain, simple cholecystectomy, and tumor debulking surgeries, are often adopted. However, the efficacy of single surgical treatment is unsatisfactory. Therefore, adjuvant therapies, including chemotherapy and radiotherapy, as well as more advanced immunotherapy and targeted therapy, are also frequently used in the comprehensive management of advanced GBC patients to improve their survival time (17).

There is currently a controversy regarding whether radical surgery should be performed for advanced GBC patients. The scope of radical surgery for advanced GBC typically includes the removal of the gallbladder, adjacent liver tissue, lymph node(s), and affected organ(s). More aggressive procedures involve pancreaticoduodenectomy (PD) and hepatopancreaticoduodenectomy (HPD) (18, 19). Some scholars argue that aggressive surgery is beneficial for patient survival (10, 20–24). A multicenter cohort study analyzed the effects of extended resection surgery on locally advanced GBC patients, confirming the role of radical resection, resulting in some patients achieving a survival time of over two years (25). Even in AJCC stage IV GBC patients, the survival rate after radical surgery has been shown to be significantly higher than that of no surgery (26, 27). Conversely, some physicians question the benefits of radical surgery, considering routine or prophylactic extended surgery to have no significant survival advantage, and non-radical surgery is more recommended (18, 28). In one report, although HPD could eradicate locally advanced GBC, it did not show superiority over non-radical surgery in terms of overall survival, complication morbidity, and mortality (28–30). Therefore, in light of the above perspectives and the findings of this study, as an alternative, non-radical surgery is adopted, as it has also been proven effective for survival in advanced GBC (19, 28, 29).

Chemotherapy is widely used in gastrointestinal tumors, and although the progression-free survival of advanced GBC is relatively short due to its particularity, it still has a positive impact (31). Currently, the gemcitabine and cisplatin (GS) regimen is the most widely accepted chemotherapy regimen for GBC (32–35). With the emergence of neoadjuvant chemotherapy, it has also created opportunities for radical surgery and longer survival time (36–40). Recently, with the use of new drugs and the conduct of more clinical trials, more chemotherapy regimens have emerged, such as FOLFOX, modified FOLFIRINOX (mFOLFIRINOX), and GEMOX regimens (35, 41). Additionally, hepatic arterial infusion chemotherapy (HAIC) is also a choice (36, 42). It is worth mentioning that even without surgery, palliative chemotherapy is beneficial for the survival of advanced GBC (43, 44).

Radiotherapy, as a treatment option, is not widely used in advanced GBC, but still has a positive role (45). Currently, radiotherapy mainly includes four forms: preoperative radiotherapy (neoadjuvant radiotherapy), intraoperative radiotherapy, postoperative radiotherapy (adjuvant radiotherapy), and palliative radiotherapy, with doses mostly concentrated in the range of 45-54 Gy (46, 47). Some scholars have pointed out that after surgery for advanced GBC, not using radiotherapy or chemoradiotherapy can increase the risk of local recurrence (47, 48). In advanced GBC, radiotherapy is usually jointly used with chemotherapy for effect enhancement, including adjuvant chemoradiotherapy and neoadjuvant chemoradiotherapy (49, 50). It is worth mentioning that for unresectable advanced GBC, both radiotherapy and chemoradiotherapy can bring survival benefits to patients and create opportunities for radical surgery (50, 51).

Immunotherapy and targeted therapy also boost the survival of advanced GBC patients (34, 52). The term immunotherapy refers mainly to immune checkpoint inhibitors, such as PD-1, PD-L1, TMB-H, and MSI/MMRd (53–56). In the phase III TOPAZ-1 trial in 2022 and the KEYNOTE-966 trial in 2023, the combination of durvalumab or pembrolizumab with gemcitabine-cisplatin demonstrated improved survival compared to treatment with gemcitabine-cisplatin alone (57–59). Additionally, several phase II clinical trials have provided evidence supporting the effectiveness of other immunotherapy regimens, such as stereotactic body radiotherapy (SBRT) combined with nivolumab and ipilimumab, camrelizumab plus FOLFOX4 or GEMOX, and nab-paclitaxel combined with sintilimab (60–64). Targeted drugs include trastuzumab, erdafitinib, lenvatinib, and so on, mainly targeting specific molecular pathways such as Hedgehog, PI3K/AKT/mTOR, Notch, ErbB, MAPK/ERK, and Angiogenesis (53, 65–67). Additionally, patient-derived tumor organoid and patient-derived tumor xenograft models can facilitate personalized treatment for patients with advanced GBC (68).

The SEER database was utilized to compare treatment models for advanced GBC patients in this study. Among all patients, through Cox regression analyses, it was observed that the “RSCR” model exhibited the most significant improvement in CSS compared to the “NSNCNR” model, with other models also showing varying degrees of improvement. After excluding treatment models with insufficient sample sizes and analyzing the post-PSM data, or after conducting analysis using only cases with complete data, the results remained robust. These results consistent with the current understanding. After subgroup analysis by AJCC stage, the efficacy of the “RSCR” model remained significant. Some discordant results observed in subgroup analyses may stem from variations in the underlying patient profiles at each AJCC stage, or from the relatively small sample sizes in certain treatment models and stage categories. It is noteworthy that the extension effect of the “NrSCR” model on CSS was also considerable, often ranking second only to the “RSCR” model. Considering the difficulty of achieving radical surgery in advanced GBC, non-radical surgery is also a treatment option for patients who cannot undergo radical surgery, of course, in conjunction with other treatment options as much as possible. Additionally, it was found that the use of radical or non-radical surgery, chemotherapy, and radiotherapy provided significant benefits to patients compared to no surgery, no chemotherapy, and no radiotherapy, and the survival time was prolonged to varying degrees after the combination of treatment options.

In our departmental cohort analysis, the absence of significant differences in clinical and demographic characteristics between the two groups (p > 0.05) indicates that the patients were comparable in terms of baseline factors at the time of treatment assignment. This comparability is crucial as it reduces the likelihood of confounding bias, allowing any observed differences in survival outcomes to be more confidently attributed to the effect of immunotherapy rather than baseline imbalances. In the KM analysis, the lack of statistical significance (p = 0.23) may be attributed to the limited sample size in our cohort, particularly with only 5 patients receiving immunotherapy. Small sample sizes often reduce the power of statistical tests, making it more difficult to detect significant differences even if a true effect exists. While the trend observed in the Kaplan-Meier curves suggests a potential benefit of immunotherapy, further studies with larger patient cohorts are needed to confirm this finding and achieve adequate statistical power. It is believed that these results can provide new insights to clinicians, indicating that when conditions permit, comprehensive treatment including surgery, chemotherapy, radiotherapy, as well as immunotherapy and targeted therapy should be provided to patients with advanced GBC, rather than limiting treatment to one or two options.

However, several limitations still exist in this innovative study. Firstly, the SEER database is a retrospective database, and data such as vital signs, nutritional status, underlying diseases of patients are not reflected in the database. Secondly, the specific location of the tumor, tumor burden, and surgical procedures were not mentioned. Additionally, the specific modes, doses, and durations of chemotherapy and radiotherapy, as well as the sequence and intervals of surgery, chemotherapy, and radiotherapy, were not within the scope of the study, and treatment options such as targeted therapy and immunotherapy were not embraced. Despite these limitations, the SEER database is still useful in terms of providing the most comprehensive data on treatment patterns and survival status in the United States to date. Additionally, to address the limitations of the database, we conducted a review of our departmental cohort and literature from the PubMed database to enhance our understanding of immunotherapy and related treatments. Based on our knowledge, a comprehensive study of all twelve treatment models selected from the SEER database is being conducted for the first time, while several studies have utilized the database to analyze GBC, none have combined such a broad range of treatment models and ranked them accordingly (69). Additionally, this study is one of the few that focuses on advanced GBC. Previous researchers have often overlooked this patient group, as advanced GBC has traditionally been considered unsuitable for surgical intervention (70). Given that the SEER database analysis is retrospective in nature, future research should incorporate more detailed clinical data, including specific surgical approaches, chemotherapy regimens, radiation doses, and the sequencing of various treatment options. Moreover, larger departmental cohorts should be established to enhance the reliability of the findings. Furthermore, we recommend that prospective studies explore these aspects in greater depth and assess their applications in clinical practice.

## 5 Conclusion

For the SEER database, the “Radical surgery + Chemotherapy + Radiotherapy” models provide the greatest survival benefit for advanced GBC patients. At the same time, the departmental cohort analysis suggests that incorporating immunotherapy may offer further advantages to patients. Providing patients with the most comprehensive treatment possible, even if the optimal treatment effect is not achieved, is a way to extend the survival of patients. As long as treatment options are taken, it is always beneficial for patient survival. This innovative finding requires more comprehensive data and prospective studies for validation.

## Data Availability

The original contributions presented in the study are included in the article/**Supplementary Material**. Further inquiries can be directed to the corresponding authors.
