# Supplementary material for: Comparison of treatment models for single primary advanced gallbladder cancer
Source: Front Immunol. 2024 Nov 13;15:1500091. doi: 10.3389/fimmu.2024.1500091 (PMC11599203; doi:10.3389/fimmu.2024.1500091)
Supplement: Supplementary file 1 [file Table1.docx]

Supplementary Material

**Comparison of treatment models for single primary advanced gallbladder cancer**

**Rongxuan Li^1†^, Xiao Chen^1†^, Bingchen Wang^1†^, Bolun Ai^2†^, Fangdi Min^3†^, Dayong Cao^1*^, Jianguo Zhou^1*^, Tao Yan^3*^**

† These authors contributed equally to this work and share first authorship

^1^ Department of Hepatobiliary Surgery, National Cancer Center/National Clinical Research Center for Cancer/Cancer Hospital, Chinese Academy of Medical Sciences and Peking Union Medical College, Beijing 100021, China

^2^ Department of Breast Surgical Oncology, National Cancer Center/National Clinical Research Center for Cancer/Cancer Hospital, Chinese Academy of Medical Sciences and Peking Union Medical College, Beijing, 100021, China

^3^ Department of Anesthesiology, National Cancer Center/National Clinical Research Center for Cancer/Cancer Hospital, Chinese Academy of Medical Sciences and Peking Union Medical College, Beijing, 100021, China

*** Correspondence:**Corresponding Author: Dayong Cao, Jianguo Zhou, Tao Yan
E-Mail: [caodayongdoctty@tom.com;](mailto:caodayongdoctty@tom.com;) [zjgtydoct@163.com;](mailto:zjgtydoct@163.com;) blizzardyt@163.com

# Supplementary Figures and Tables

## Supplementary Figures


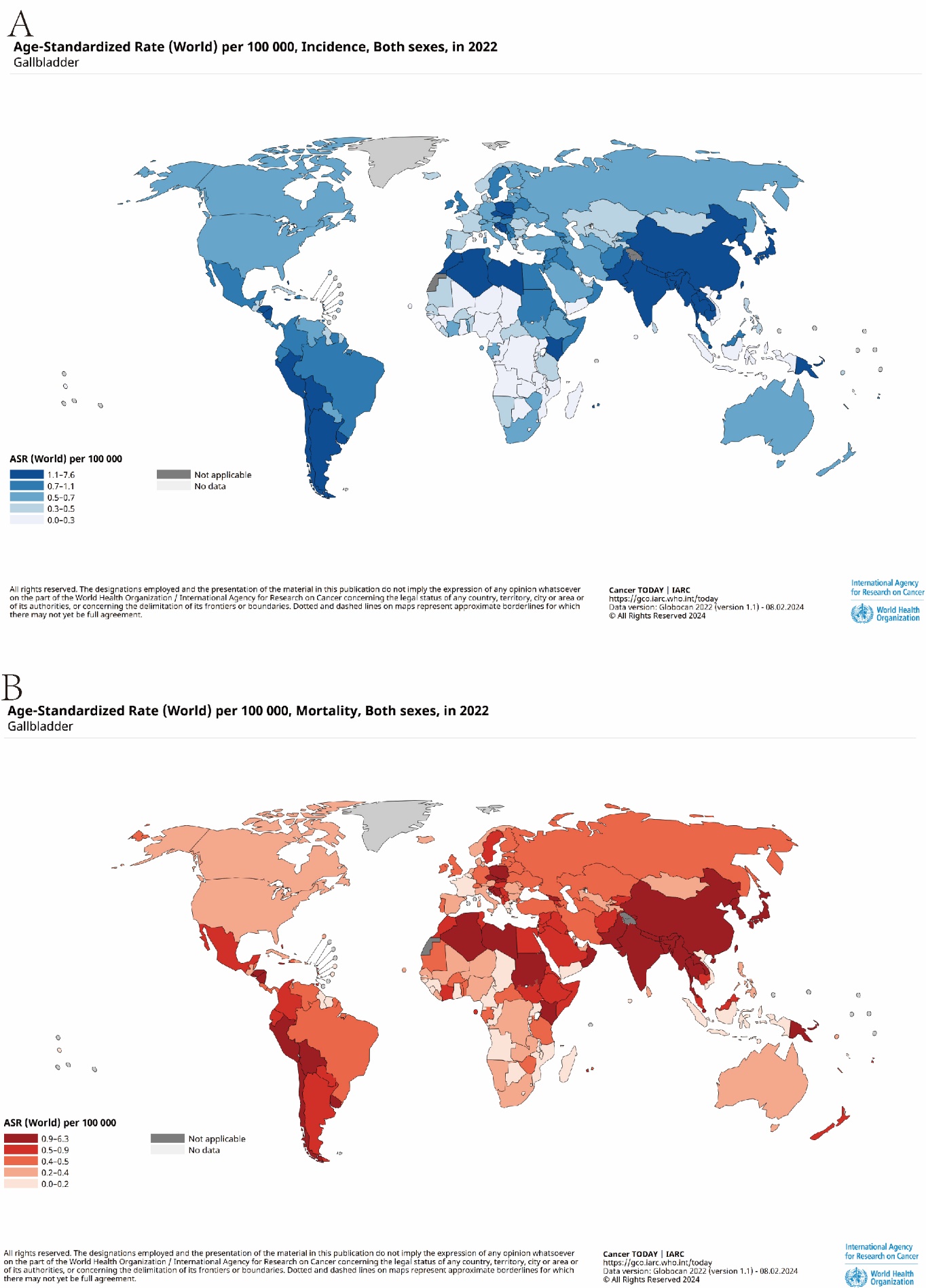


**Supplementary Figure 1.** Global map depicting GBC by country in terms of age-standardized incidence rate and age-standardized mortality rate in 2022. A: Age-standardized incidence rate; B: Age-standardized mortality rate. ASR: age-standardized rate. Data from: https://gco.iarc.fr/en .

**
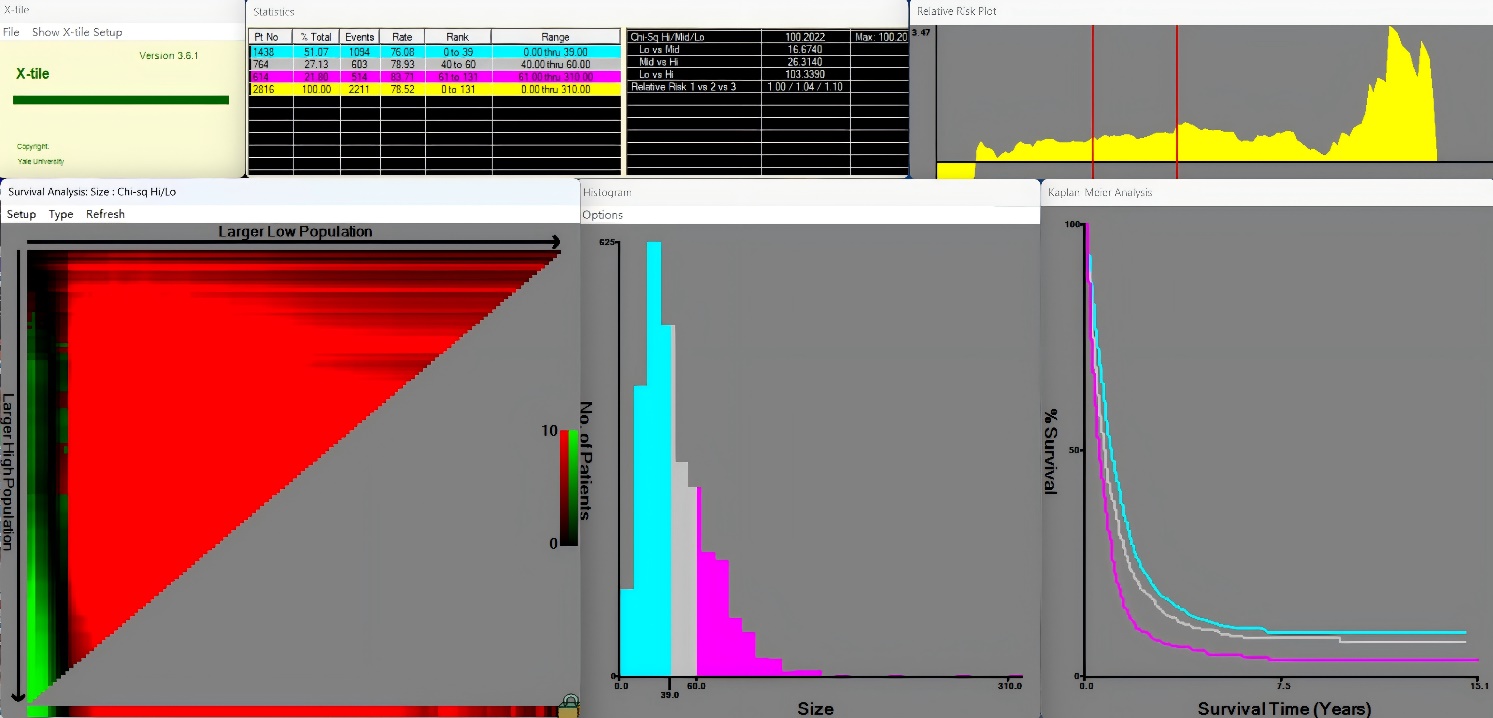
**

**Supplementary Figure 2.** The optimal cutoff points of tumor size, measured in millimeters (mm), were determined using X-tile software (version 3.6.1).


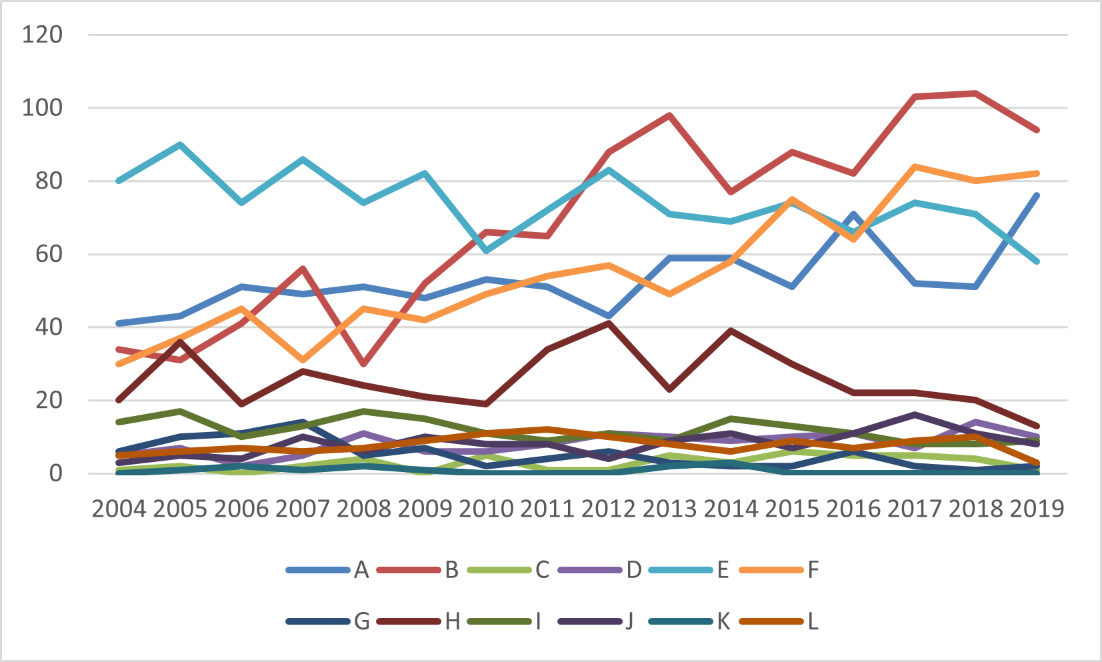


**Supplementary Figure 3.** The changes in the selection of various treatment models over time.


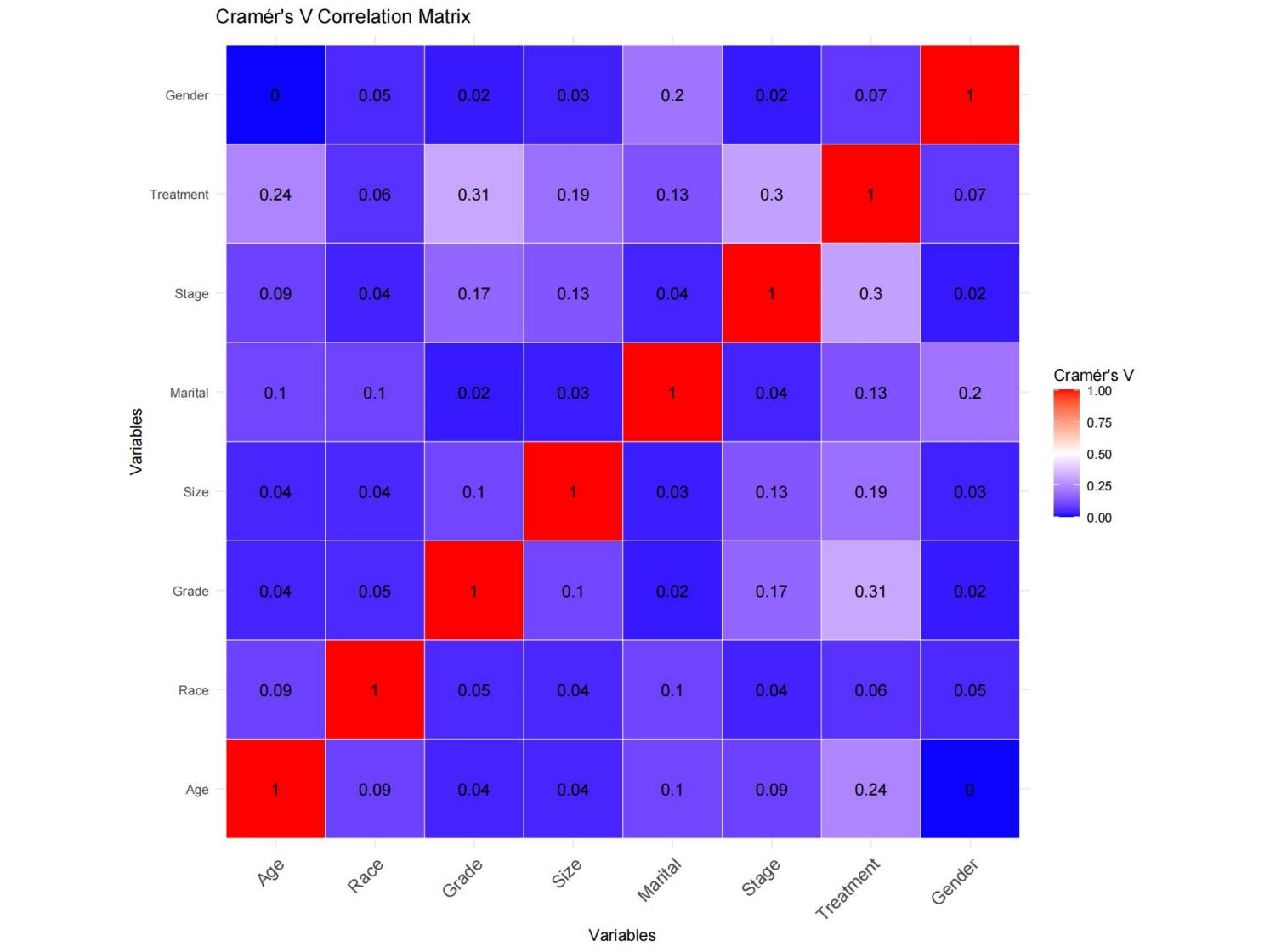


**Supplementary Figure 4.** The correlation between covariates, with the results presented in a matrix format.

## Supplementary Tables

|  | Age | Gender | Size | Grade | Stage | Surgery | Chemotherapy | Radiotherapy | Immunotherapy |
| --- | --- | --- | --- | --- | --- | --- | --- | --- | --- |
| 1 | <65 | Female | Unknown | Grade III | IIIB | Radical surgery | Yes | No | No |
| 2 | <65 | Male | ≥61 | Grade III | IIIA | Radical surgery | Yes | No | Yes (PD-1) |
| 3 | <65 | Male | Unknown | Grade I | IIIB | Non-radical surgery | Yes | No | No |
| 4 | <65 | Female | ≤39 | Grade III | IVB | Radical surgery | Yes | No | No |
| 5 | <65 | Male | Unknown | Grade III | IVB | Non-radical surgery | Yes | No | Yes (PD-1) |
| 6 | <65 | Male | ≤39 | Grade II | IVB | Radical surgery | Yes | No | No |
| 7 | ≥65 | Male | 40-60 | Grade III | IVB | Radical surgery | No | No | No |
| 8 | <65 | Female | ≤39 | Grade III | IIIB | Radical surgery | No | No | No |
| 9 | <65 | Male | ≤39 | Grade III | IVB | Non-radical surgery | No | No | No |
| 10 | <65 | Male | ≤39 | Grade II | IIIA | Radical surgery | No | No | No |
| 11 | <65 | Female | 40-60 | Grade III | IVB | Radical surgery | Yes | No | Yes (PD-1) |
| 12 | <65 | Female | ≥61 | Grade III | IIIB | Radical surgery | Yes | No | No |
| 13 | <65 | Female | ≤39 | Grade III | IIIA | Radical surgery | Yes | No | Yes (PD-L1) |
| 14 | ≥65 | Female | ≤39 | Grade III | IIIB | Radical surgery | Yes | No | No |
| 15 | ≥65 | Male | 40-60 | Grade III | IIIB | Radical surgery | Yes | Yes | Yes (PD-1) |

**Supplementary Table 1.** The basic information of patients in the departmental cohort.

|  | Univariable | | | | | Multivariable | | | | |
| --- | --- | --- | --- | --- | --- | --- | --- | --- | --- | --- |
| Characteristic | N | Event N | HR^1^ | 95% CI^1^ | p-value | N | Event N | HR^1^ | 95% CI^1^ | p-value |
| Age |  |  |  |  |  |  |  |  |  |  |
| ＜65 | 1,971 | 1,588 | reference | reference |  | 1,971 | 1,588 | reference | reference |  |
| ≥65 | 3,126 | 2,586 | 1.22 | 1.15, 1.30 | <0.001 | 3,126 | 2,586 | 1.15 | 1.07, 1.22 | <0.001 |
| Gender |  |  |  |  |  |  |  |  |  |  |
| Female | 3,601 | 2,920 | reference | reference |  | 3,601 | 2,920 | reference | reference |  |
| Male | 1,496 | 1,254 | 1.1 | 1.03, 1.17 | 0.005 | 1,496 | 1,254 | 1.12 | 1.04, 1.20 | 0.001 |
| Marital |  |  |  |  |  |  |  |  |  |  |
| Yes | 2,649 | 2,161 | reference | reference |  | 2,649 | 2,161 | reference | reference |  |
| No | 2,280 | 1,876 | 1.17 | 1.10, 1.24 | <0.001 | 2,280 | 1,876 | 1.09 | 1.02, 1.16 | 0.007 |
| Unknown | 168 | 137 | 1.12 | 0.94, 1.33 | 0.21 | 168 | 137 | 1.19 | 1.00, 1.42 | 0.049 |
| Size |  |  |  |  |  |  |  |  |  |  |
| ≤39 | 1,426 | 1,083 | reference | reference |  | 1,426 | 1,083 | reference | reference |  |
| 40-60 | 754 | 595 | 1.21 | 1.10, 1.34 | <0.001 | 754 | 595 | 1.13 | 1.02, 1.25 | 0.022 |
| ≥61 | 607 | 507 | 1.67 | 1.50, 1.86 | <0.001 | 607 | 507 | 1.39 | 1.25, 1.55 | <0.001 |
| Unknown | 2,310 | 1,989 | 1.65 | 1.53, 1.77 | <0.001 | 2,310 | 1,989 | 1.23 | 1.14, 1.33 | <0.001 |
| Grade |  |  |  |  |  |  |  |  |  |  |
| Grade I | 248 | 194 | reference | reference |  | 248 | 194 | reference | reference |  |
| Grade II | 1,264 | 1,037 | 1.17 | 1.00, 1.36 | 0.051 | 1,264 | 1,037 | 1.25 | 1.08, 1.46 | 0.004 |
| Grade III | 1,413 | 1,227 | 1.68 | 1.44, 1.95 | <0.001 | 1,413 | 1,227 | 1.73 | 1.48, 2.01 | <0.001 |
| Grade IV | 47 | 40 | 1.96 | 1.40, 2.76 | <0.001 | 47 | 40 | 2.27 | 1.61, 3.20 | <0.001 |
| Unknown | 2,125 | 1,676 | 2.2 | 1.89, 2.55 | <0.001 | 2,125 | 1,676 | 1.39 | 1.19, 1.63 | <0.001 |
| Stage |  |  |  |  |  |  |  |  |  |  |
| IIIA | 1,245 | 981 | reference | reference |  | 1,245 | 981 | reference | reference |  |
| IIIB | 802 | 514 | 0.56 | 0.51, 0.63 | <0.001 | 802 | 514 | 0.73 | 0.66, 0.82 | <0.001 |
| IVA | 163 | 140 | 1.34 | 1.12, 1.60 | 0.001 | 163 | 140 | 1.3 | 1.09, 1.56 | 0.004 |
| IVB | 2,887 | 2,539 | 1.77 | 1.64, 1.91 | <0.001 | 2,887 | 2,539 | 1.74 | 1.60, 1.88 | <0.001 |
| Treatment |  |  |  |  |  |  |  |  |  |  |
| A | 849 | 765 | reference | reference |  | 849 | 765 | reference | reference |  |
| B | 1,109 | 968 | 0.46 | 0.41, 0.50 | <0.001 | 1,109 | 968 | 0.44 | 0.39, 0.48 | <0.001 |
| D | 132 | 117 | 0.4 | 0.33, 0.48 | <0.001 | 132 | 117 | 0.42 | 0.34, 0.51 | <0.001 |
| E | 1,185 | 955 | 0.34 | 0.31, 0.38 | <0.001 | 1,185 | 955 | 0.49 | 0.43, 0.54 | <0.001 |
| F | 882 | 677 | 0.24 | 0.22, 0.27 | <0.001 | 882 | 677 | 0.3 | 0.26, 0.33 | <0.001 |
| G | 83 | 69 | 0.26 | 0.20, 0.33 | <0.001 | 83 | 69 | 0.38 | 0.29, 0.49 | <0.001 |
| H | 411 | 299 | 0.15 | 0.13, 0.18 | <0.001 | 411 | 299 | 0.24 | 0.21, 0.28 | <0.001 |
| I | 190 | 141 | 0.27 | 0.23, 0.33 | <0.001 | 190 | 141 | 0.37 | 0.31, 0.45 | <0.001 |
| J | 131 | 93 | 0.21 | 0.17, 0.26 | <0.001 | 131 | 93 | 0.25 | 0.20, 0.31 | <0.001 |
| L | 125 | 90 | 0.15 | 0.12, 0.19 | <0.001 | 125 | 90 | 0.24 | 0.19, 0.30 | <0.001 |
| ^1^HR = Hazard Ratio, CI = Confidence Interval | | | | | | | | | | |

**Supplementary Table 2.** Univariate and multivariate Cox proportional hazards models of CSS for advanced GBC patients after excluding models with insufficient sample sizes (<1%).

| Characteristic | Surgery | | p-value |
| --- | --- | --- | --- |
|  | Non-radical, N = 427^1^ | Radical, N = 427^1^ |  |
| Age |  |  | 0.945^2^ |
| ＜65 | 186 (43.56%) | 185 (43.33%) |  |
| ≥65 | 241 (56.44%) | 242 (56.67%) |  |
| Gender |  |  | 0.769^2^ |
| Female | 288 (67.45%) | 292 (68.38%) |  |
| Male | 139 (32.55%) | 135 (31.62%) |  |
| Marital |  |  | 0.946^2^ |
| Yes | 237 (55.50%) | 239 (55.97%) |  |
| No | 184 (43.09%) | 181 (42.39%) |  |
| Unknown | 6 (1.41%) | 7 (1.64%) |  |
| Size |  |  | 0.916^2^ |
| ≤39 | 150 (35.13%) | 141 (33.02%) |  |
| 40-60 | 100 (23.42%) | 101 (23.65%) |  |
| ≥61 | 63 (14.75%) | 68 (15.93%) |  |
| Unknown | 114 (26.70%) | 117 (27.40%) |  |
| Grade |  |  | 0.979^2^ |
| Grade I | 25 (5.85%) | 24 (5.62%) |  |
| Grade II | 171 (40.05%) | 169 (39.58%) |  |
| Grade III | 158 (37.00%) | 158 (37.00%) |  |
| Grade IV | 4 (0.94%) | 6 (1.41%) |  |
| Unknown | 69 (16.16%) | 70 (16.39%) |  |
| Stage |  |  | 0.821^2^ |
| IIIA | 106 (24.82%) | 117 (27.40%) |  |
| IIIB | 138 (32.32%) | 137 (32.08%) |  |
| IVA | 12 (2.81%) | 10 (2.34%) |  |
| IVB | 171 (40.05%) | 163 (38.17%) |  |
| Radiation |  |  | 0.940^2^ |
| Yes | 121 (28.34%) | 122 (28.57%) |  |
| No | 306 (71.66%) | 305 (71.43%) |  |
| Chemotherapy |  |  | 0.535^2^ |
| Yes | 244 (57.14%) | 235 (55.04%) |  |
| No | 183 (42.86%) | 192 (44.96%) |  |
| ^1^n (%) | | | |
| ^2^Pearson's Chi-squared test | | | |

**Supplementary Table 3.** Demographic and clinical characteristics for patients undergoing non-radical surgery and radical surgery after PSM.

|  | Univariable | | | | | Multivariable | | | | |
| --- | --- | --- | --- | --- | --- | --- | --- | --- | --- | --- |
| Characteristic | N | Event N | HR^1^ | 95% CI^1^ | p-value | N | Event N | HR^1^ | 95% CI^1^ | p-value |
| Age |  |  |  |  |  |  |  |  |  |  |
| ＜65 | 371 | 264 | reference | reference |  | 371 | 264 | reference | reference |  |
| ≥65 | 483 | 371 | 1.24 | 1.06, 1.46 | 0.007 | 483 | 371 | 1.18 | 1.00, 1.39 | 0.053 |
| Gender |  |  |  |  |  |  |  |  |  |  |
| Female | 580 | 428 | reference | reference |  | 580 | 428 | reference | reference |  |
| Male | 274 | 207 | 1.07 | 0.90, 1.26 | 0.452 | 274 | 207 | 1.16 | 0.97, 1.38 | 0.101 |
| Marital |  |  |  |  |  |  |  |  |  |  |
| Yes | 476 | 349 | reference | reference |  | 476 | 349 | reference | reference |  |
| No | 365 | 276 | 1.28 | 1.09, 1.50 | 0.002 | 365 | 276 | 1.11 | 0.94, 1.32 | 0.208 |
| Unknown | 13 | 10 | 1.31 | 0.70, 2.45 | 0.403 | 13 | 10 | 1.82 | 0.94, 3.49 | 0.074 |
| Size |  |  |  |  |  |  |  |  |  |  |
| ≤39 | 291 | 210 | reference | reference |  | 291 | 210 | reference | reference |  |
| 40-60 | 201 | 144 | 1.14 | 0.92, 1.41 | 0.222 | 201 | 144 | 1.02 | 0.82, 1.27 | 0.868 |
| ≥61 | 131 | 103 | 1.9 | 1.49, 2.40 | <0.001 | 131 | 103 | 1.56 | 1.22, 1.99 | <0.001 |
| Unknown | 231 | 178 | 1.45 | 1.19, 1.77 | <0.001 | 231 | 178 | 1.23 | 1.00, 1.51 | 0.055 |
| Grade |  |  |  |  |  |  |  |  |  |  |
| Grade I | 49 | 38 | reference | reference |  | 49 | 38 | reference | reference |  |
| Grade II | 340 | 266 | 1.06 | 0.75, 1.49 | 0.742 | 340 | 266 | 1.13 | 0.80, 1.59 | 0.487 |
| Grade III | 316 | 261 | 1.46 | 1.04, 2.05 | 0.031 | 316 | 261 | 1.56 | 1.11, 2.20 | 0.011 |
| Grade IV | 10 | 8 | 3.49 | 1.62, 7.52 | 0.001 | 10 | 8 | 3.9 | 1.80, 8.47 | <0.001 |
| Unknown | 139 | 62 | 0.94 | 0.63, 1.41 | 0.757 | 139 | 62 | 0.97 | 0.65, 1.47 | 0.898 |
| Stage |  |  |  |  |  |  |  |  |  |  |
| IIIA | 223 | 157 | reference | reference |  | 223 | 157 | reference | reference |  |
| IIIB | 275 | 176 | 0.78 | 0.63, 0.97 | 0.028 | 275 | 176 | 0.86 | 0.69, 1.07 | 0.173 |
| IVA | 22 | 19 | 1.62 | 1.01, 2.61 | 0.048 | 22 | 19 | 1.86 | 1.14, 3.02 | 0.013 |
| IVB | 334 | 283 | 1.86 | 1.53, 2.27 | <0.001 | 334 | 283 | 2 | 1.62, 2.47 | <0.001 |
| Surgery |  |  |  |  |  |  |  |  |  |  |
| Non-radical | 427 | 327 | reference | reference |  | 427 | 327 | reference | reference |  |
| Radical | 427 | 308 | 0.87 | 0.75, 1.02 | 0.083 | 427 | 308 | 0.81 | 0.69, 0.95 | 0.008 |
| Radiation |  |  |  |  |  |  |  |  |  |  |
| yes | 243 | 168 | reference | reference |  | 243 | 168 | reference | reference |  |
| no | 611 | 467 | 1.92 | 1.61, 2.29 | <0.001 | 611 | 467 | 1.18 | 0.96, 1.45 | 0.115 |
| Chemotherapy |  |  |  |  |  |  |  |  |  |  |
| yes | 479 | 336 | reference | reference |  | 479 | 336 | reference | reference |  |
| no | 375 | 299 | 1.85 | 1.58, 2.16 | <0.001 | 375 | 299 | 1.74 | 1.45, 2.09 | <0.001 |
| ^1^HR = Hazard Ratio, CI = Confidence Interval | | | | | | | | | | |

**Supplementary Table 4.** Univariable and multivariable Cox proportional hazards models for CSS in advanced GBC patients, conducted after PSM based on the type of surgery.

|  | Univariable | | | | | Multivariable | | | | |
| --- | --- | --- | --- | --- | --- | --- | --- | --- | --- | --- |
| Characteristic | N | Event N | HR^1^ | 95% CI^1^ | p-value | N | Event N | HR^1^ | 95% CI^1^ | p-value |
| Age |  |  |  |  |  |  |  |  |  |  |
| ＜65 | 673 | 539 | reference | reference |  | 673 | 539 | reference | reference |  |
| ≥65 | 1,089 | 910 | 1.29 | 1.16, 1.44 | <0.001 | 1,089 | 910 | 1.25 | 1.12, 1.40 | <0.001 |
| Gender |  |  |  |  |  |  |  |  |  |  |
| Female | 1,246 | 1,013 | reference | reference |  | 1,246 | 1,013 | reference | reference |  |
| Male | 516 | 436 | 1.14 | 1.01, 1.27 | 0.027 | 516 | 436 | 1.2 | 1.07, 1.35 | 0.002 |
| Marital |  |  |  |  |  |  |  |  |  |  |
| Yes | 952 | 770 | reference | reference |  | 952 | 770 | reference | reference |  |
| No | 810 | 679 | 1.23 | 1.11, 1.36 | <0.001 | 810 | 679 | 1.12 | 1.00, 1.25 | 0.042 |
| Size |  |  |  |  |  |  |  |  |  |  |
| ≤39 | 966 | 780 | reference | reference |  | 966 | 780 | reference | reference |  |
| 40-60 | 475 | 391 | 1.17 | 1.04, 1.32 | 0.011 | 475 | 391 | 1.09 | 0.96, 1.23 | 0.185 |
| ≥61 | 321 | 278 | 1.61 | 1.40, 1.85 | <0.001 | 321 | 278 | 1.37 | 1.19, 1.58 | <0.001 |
| Grade |  |  |  |  |  |  |  |  |  |  |
| Grade I | 129 | 99 | reference | reference |  | 129 | 99 | reference | reference |  |
| Grade II | 754 | 601 | 1.11 | 0.90, 1.38 | 0.324 | 754 | 601 | 1.12 | 0.91, 1.39 | 0.293 |
| Grade III | 850 | 724 | 1.58 | 1.28, 1.95 | <0.001 | 850 | 724 | 1.54 | 1.24, 1.91 | <0.001 |
| Grade IV | 29 | 25 | 2.12 | 1.36, 3.28 | <0.001 | 29 | 25 | 2.16 | 1.39, 3.36 | <0.001 |
| Stage |  |  |  |  |  |  |  |  |  |  |
| IIIA | 529 | 414 | reference | reference |  | 529 | 414 | reference | reference |  |
| IIIB | 452 | 317 | 0.68 | 0.58, 0.78 | <0.001 | 452 | 317 | 0.8 | 0.69, 0.93 | 0.003 |
| IVA | 58 | 50 | 1.52 | 1.13, 2.04 | 0.005 | 58 | 50 | 1.44 | 1.06, 1.97 | 0.022 |
| IVB | 723 | 668 | 1.9 | 1.67, 2.15 | <0.001 | 723 | 668 | 2.02 | 1.77, 2.31 | <0.001 |
| Treatment |  |  |  |  |  |  |  |  |  |  |
| A | 60 | 58 | reference | reference |  | 60 | 58 | reference | reference |  |
| B | 110 | 104 | 0.43 | 0.31, 0.59 | <0.001 | 110 | 104 | 0.46 | 0.33, 0.63 | <0.001 |
| C | 7 | 6 | 0.28 | 0.12, 0.65 | 0.003 | 7 | 6 | 0.32 | 0.14, 0.74 | 0.008 |
| D | 20 | 20 | 0.43 | 0.26, 0.71 | 0.001 | 20 | 20 | 0.45 | 0.27, 0.76 | 0.003 |
| E | 592 | 484 | 0.27 | 0.20, 0.35 | <0.001 | 592 | 484 | 0.46 | 0.35, 0.62 | <0.001 |
| F | 414 | 345 | 0.2 | 0.15, 0.27 | <0.001 | 414 | 345 | 0.29 | 0.22, 0.39 | <0.001 |
| G | 39 | 32 | 0.19 | 0.12, 0.30 | <0.001 | 39 | 32 | 0.34 | 0.22, 0.54 | <0.001 |
| H | 238 | 177 | 0.12 | 0.09, 0.17 | <0.001 | 238 | 177 | 0.24 | 0.17, 0.33 | <0.001 |
| I | 116 | 94 | 0.25 | 0.18, 0.35 | <0.001 | 116 | 94 | 0.4 | 0.29, 0.56 | <0.001 |
| J | 77 | 60 | 0.17 | 0.12, 0.25 | <0.001 | 77 | 60 | 0.23 | 0.16, 0.34 | <0.001 |
| K | 9 | 8 | 0.19 | 0.09, 0.40 | <0.001 | 9 | 8 | 0.38 | 0.18, 0.80 | 0.011 |
| L | 80 | 61 | 0.13 | 0.09, 0.18 | <0.001 | 80 | 61 | 0.24 | 0.17, 0.35 | <0.001 |
| ^1^HR = Hazard Ratio, CI = Confidence Interval | | | | | | | | | | |

**Supplementary Table 5.** Univariate and multivariate Cox proportional hazards models of CSS for advanced GBC patients after excluding models with incomplete data.

| Characteristic | Immunotherapy | | p-value |
| --- | --- | --- | --- |
|  | Yes, N = 5^1^ | No, N = 10^1^ |  |
| Age |  |  | >0.999^2^ |
| ˂65 | 4 (80.00%) | 8 (80.00%) |  |
| ≥65 | 1 (20.00%) | 2 (20.00%) |  |
| Gender |  |  | >0.999^2^ |
| Female | 2 (40.00%) | 5 (50.00%) |  |
| Male | 3 (60.00%) | 5 (50.00%) |  |
| Size |  |  | 0.503^2^ |
| ≤39 | 1 (20.00%) | 6 (60.00%) |  |
| 40-60 | 2 (40.00%) | 1 (10.00%) |  |
| ≥61 | 1 (20.00%) | 1 (10.00%) |  |
| Unknown | 1 (20.00%) | 2 (20.00%) |  |
| Grade |  |  | 0.670^2^ |
| Grade I | 0 (0.00%) | 1 (10.00%) |  |
| Grade II | 0 (0.00%) | 2 (20.00%) |  |
| Grade III | 5 (100.00%) | 7 (70.00%) |  |
| Stage |  |  | 0.336^2^ |
| IIIA | 2 (40.00%) | 1 (10.00%) |  |
| IIIB | 1 (20.00%) | 5 (50.00%) |  |
| IVB | 2 (40.00%) | 4 (40.00%) |  |
| Surgery |  |  | >0.999^2^ |
| Non-radical surgery | 1 (20.00%) | 2 (20.00%) |  |
| Radical surgery | 4 (80.00%) | 8 (80.00%) |  |
| Chemotherapy |  |  | 0.231^2^ |
| Yes | 5 (100.00%) | 6 (60.00%) |  |
| No | 0 (0.00%) | 4 (40.00%) |  |
| Radiotherapy |  |  | 0.333^2^ |
| Yes | 1 (20.00%) | 0 (0.00%) |  |
| No | 4 (80.00%) | 10 (100.00%) |  |
| ^1^n (%) | | | |
| ^2^Fisher's exact test | | | |

**Supplementary Table 6.** Demographic and clinical characteristics of patients with advanced GBC in the departmental cohort.
